# Dietary supplementation of zinc oxide modulates intestinal functionality during the post-weaning period in clinically healthy piglets

**DOI:** 10.1186/s40104-023-00925-1

**Published:** 2023-10-04

**Authors:** Dirkjan Schokker, Soumya K. Kar, Els Willems, Alex Bossers, Ruud A. Dekker, Alfons J. M. Jansman

**Affiliations:** 1 Wageningen Bioveterinary Research, Lelystad, The Netherlands; 2 Wageningen Livestock Research, Wageningen University & Research, Wageningen, The Netherlands; 3 Royal Agrifirm Group, Apeldoorn, The Netherlands; 4 Institute for Risk Assessment Sciences, Utrecht University, Utrecht, The Netherlands

**Keywords:** Immune system, Intestinal functionality, Microbiota, Piglets, Zinc oxide

## Abstract

**Background:**

To improve our understanding of host and intestinal microbiome interaction, this research investigated the effects of a high-level zinc oxide in the diet as model intervention on the intestinal microbiome and small intestinal functionality in clinically healthy post-weaning piglets. In study 1, piglets received either a high concentration of zinc (Zn) as zinc oxide (ZnO, Zn, 2,690 mg/kg) or a low Zn concentration (100 mg/kg) in the diet during the post weaning period (d 14–23). The effects on the piglet’s small intestinal microbiome and functionality of intestinal tissue were investigated. In study 2, the impact of timing of the dietary zinc intervention was investigated, i.e., between d 0–14 and/or d 14–23 post weaning, and the consecutive effects on the piglet’s intestinal functionality, here referring to microbiota composition and diversity and gene expression profiles.

**Results:**

Differences in the small intestinal functionality were observed during the post weaning period between piglets receiving a diet with a low or high concentration ZnO content. A shift in the microbiota composition in the small intestine was observed that could be characterized as a non-pathological change, where mainly the commensals inter-changed. In the immediate post weaning period, i.e., d 0–14, the highest number of differentially expressed genes (DEGs) in intestinal tissue were observed between animals receiving a diet with a low or high concentration ZnO content, i.e., 23 DEGs in jejunal tissue and 11 DEGs in ileal tissue. These genes are involved in biological processes related to immunity and inflammatory responses. For example, genes *CD59* and *REG3G* were downregulated in the animals receiving a diet with a high concentration ZnO content compared to low ZnO content in both jejunum and ileum tissue. In the second study, a similar result was obtained regarding the expression of genes in intestinal tissue related to immune pathways when comparing piglets receiving a diet with a high concentration ZnO content compared to low ZnO content.

**Conclusions:**

Supplementing a diet with a pharmaceutical level of Zn as ZnO for clinically healthy post weaning piglets influences various aspects intestinal functionality, in particular in the first two weeks post-weaning. The model intervention increased both the alpha diversity of the intestinal microbiome and the expression of a limited number of genes linked to the local immune system in intestinal tissue. The effects do not seem related to a direct antimicrobial effect of ZnO.

**Supplementary Information:**

The online version contains supplementary material available at 10.1186/s40104-023-00925-1.

## Background

The health status of pigs is determined by both the competence and responses of the immune system, both together shaping the resilience of pigs towards environmental stressors and pathogenic challenges [1]. Studies have shown that many factors can influence the host-microbiota interactions in the gastrointestinal tract [2, 3], including genetics [4], environment [5], and nutrition [6, 7].

Dietary supplementation of zinc oxide (ZnO) in piglets is well studied in relation to its effects on gastro-intestinal health in the post-weaning phase and prevention of post-weaning diarrhoea and effects on the host-microbiota interactions in the gut. Inclusion of pharmaceutical levels of Zn (> 2,500 mg/kg of Zn, equivalent to approximately 3,100 mg/kg of ZnO) in the diet were shown to have beneficial effects on health, resilience, and performance in piglets [8–15]. ZnO is not an alternative to either growth promoting, as used in the past, or to curative antibiotics, but might reduce the need for use of therapeutic antibiotics in post-weaning piglets. It should be noted that the use of a high concentration of ZnO in the diet for pigs at pharmaceutical level has been prohibited in the EU from June 2022 onwards [16, 17]. Nevertheless, evaluation of the effects of ZnO as modulator of the intestinal microbiome and potential modulator of intestinal functionality during the post-weaning period of pigs can still serve as a model or case study for studying dietary interventions. The proposed mode of action is multitude and appears to be linked to increased nutrient absorption/digestibility and intestinal morphology [18], other beneficial effects have been hypothesized for the immune system and intestinal integrity [19]. For example, high dietary inclusion of ZnO (2,500–3,000 mg/kg) has the capacity to change the microbial composition in the small intestine immediately post-weaning period [14, 20], and reduces the expression of inflammatory genes in gut tissue after a challenge with an enterotoxigenic *E. coli* (ETEC) [21]. A similar effect of inclusion of ZnO in the diet influenced the expression of proteins in gut tissue of post-weaning piglets that are involved in oxidative stress, cell differentiation, and apoptosis [22]. Taken together, these studies showed that the use of zinc oxide in the diet of post-weaning piglets modulates both intestinal health and functionality.

The age of pigs is another factor that has a large impact on intestinal functionality. At farrowing, the microbial colonization of the gut starts immediately, and the microbial composition continues to change until weaning in a highly dynamic way, referred to as microbial succession. The process can be influenced by e.g., antibiotic treatment [23–25] and by diet ingredient and nutrient composition [26, 27]. Gut development encompasses the enlargement of the absorptive surface [28], the advancement of gut barrier functionality [29], and maturation of the local immune system [30]. For the establishment of a well-balanced gut ecosystem, both farrowing and weaning transitions showed a shift in the intestinal microbiota composition [6, 31]. Furthermore, modulation of the development of the local and systemic immune system is possible via the intestinal microbiome. Effects of early life dietary interventions and their instant and long-term effects is an increasing area of research in both the human and pig domain [32, 33]. The former shows the essential role of the gut as gatekeeper of health. By improvement of our understanding of underlying processes linked to intestinal functionality, further possibilities arise to support health of pigs via dietary interventions which could contribute to a more sustainable pig production in the future.

The overall aim of the present work is to obtain more information of the impact of dietary interventions on intestinal functionality in clinically healthy post-weaning piglets using dietary ZnO supplementation as a model. Two studies were conducted in chronological order to: 1) decipher the effects of post-weaning supplementation of pharmaceutical levels of ZnO on the small intestinal microbiome and intestinal functionality, and 2) observe the effects of the timing of post-weaning dietary supplementation of pharmaceutical ZnO on the small intestinal microbiome and intestinal functionality in clinically healthy piglets.

## Methods

In these studies, ZnO was used as a model dietary intervention to investigate the effects in clinically healthy piglets. To this end, we have designed two studies in a chronological order. Where study 1 focused on the effect of post-weaning supplementation of pharmaceutical levels of ZnO on intestinal microbiome and functionality. This window is similar to the one frequently used in practice to administer ZnO to prevent or combat PW diarrhoea. The primary focus of study 2 was on the effect of timing of the ZnO supplementation on intestinal microbiome and functionality. Study 2 also contained similar treatment groups as in study 1 (d 0–14 PW administration), which may serve as validation of the observed results from study 1.

### Study abbreviations

**Fig. 1:**
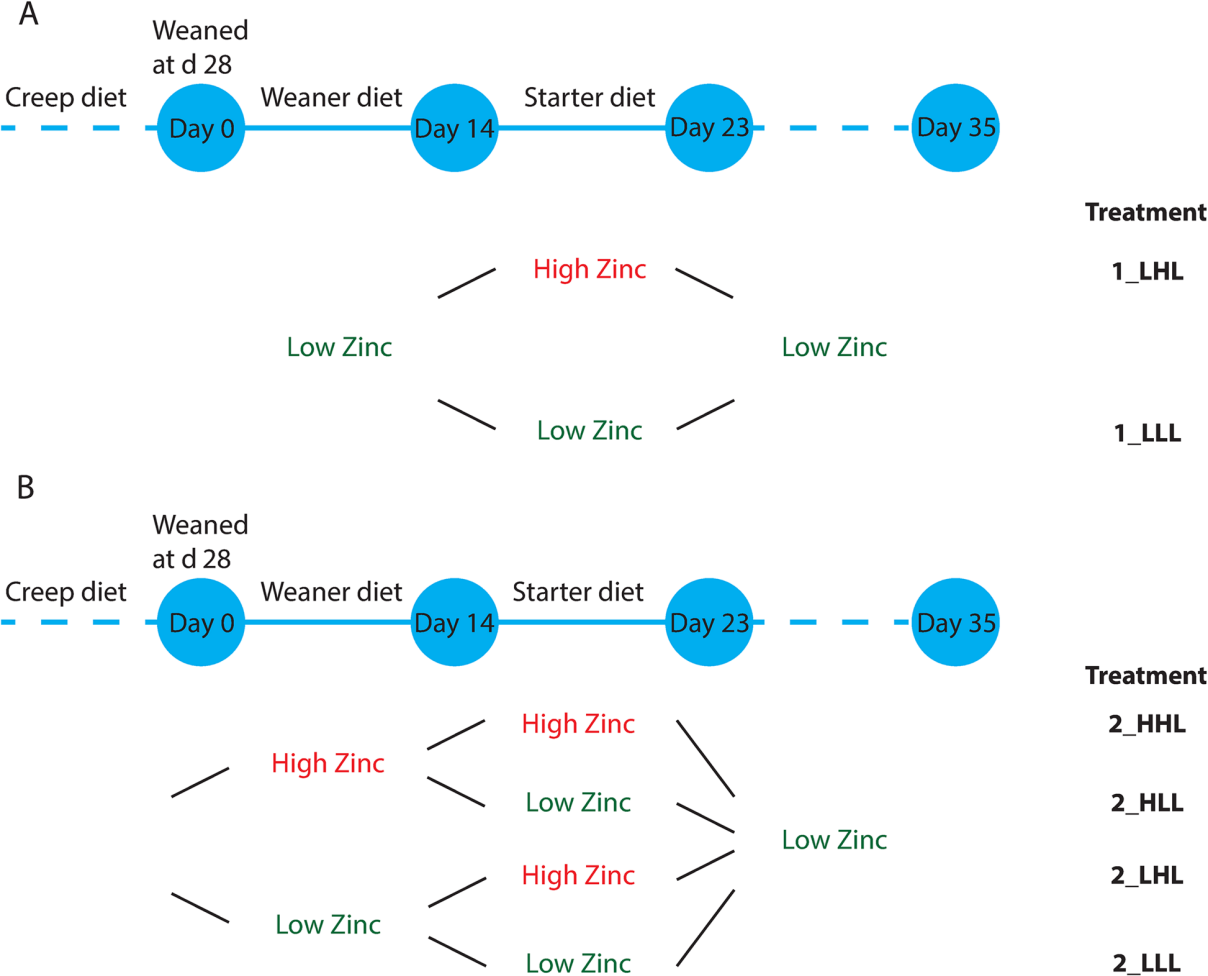
Schematic representation of the experimental designs of study 1 and 2. In the studies, the piglets received a low or high Zn diet in weaner and/or starter diets. Panel A shows the design of the first study, with two dietary treatments, i.e., 1_LHL and 1_LLL. Panel B shows the design of the second study, which evaluated four dietary treatments, i.e., 2_LLL, 2_LHL, 2_HLL, and 2_HHL

The diets were provided containing either a regular concentration of Zn (100 mg/kg) or a pharmaceutical level of Zn concentration (2,690 mg/kg), where the regular concentration was defined as low (L) and pharmaceutical levels as high (H). To indicate experimental treatments, we used first the number of the study (1 or 2), followed by an underscore and a letter indicating the dietary concentration of ZnO applied in the consecutive periods post weaning (d 0–14, d 14–23 and d 23–35). Thus, 1_LLL refers to study 1, group that received low zinc during all three phases (d 0–35), and 1_LHL refers to study 1, group that received high zinc during second phase (d 14–23). For the study 2, 2_HLL refers to study 2, group that received high zinc during first phase (d 0–14); 2_HHL refers to study 2, group that received high zinc during first and second phase (d 0–23); 2_LLL refers to study 2, group that received high zinc during all three phases (d 0–35); and 2_LHL refers to study 2, group that received high zinc during second phase (d 14–23). The experimental design for both studies is schematically presented in Fig. 1.

### Study 1

#### Experimental design, housing and feeding

The study was performed in the experimental facilities of Agrifirm, Laverdonk in Heeswijk-Dinther, the Netherlands. This animal experiment was approved by the institutional animal experiment committee under code 2013095.b, in accordance with the Dutch regulations on animal experiments.

The piglets were of the Tempo × Topigs 20 genotype. At the start of the study, the piglets were allocated per pen in a way that the mean body weight per pen was 7.9 ± 0.8 kg. Male (boars) and female piglets were equally distributed over pens and litter mates were divided equally over pens as far as possible. The piglets were weaned at a mean age of 28 ± 1.5 d. The piglets received creep feed during the suckling period. The piglets were housed in floor pens (1.75 m × 3.00 m) with 12 piglets per pen. The piglets were fed ad libitum using a dry feed dispenser and the diets were provided as pellets. The animals had free access to water via an automatic drinking device. The ingredient and calculated nutrient composition of the weaning diet and the starter diet (control) are provided in Table S1. The diets were formulated to be nutritionally adequate using data on the composition and nutritional value of feed ingredients [34]. After weaning, the piglets were fed the same weaning diet during the period of d 0 till 14. From d 14 till 23 after weaning, the experimental starter diets were provided containing either a regular or a high Zn concentration (analysed Zn concentrations 100 and 2,690 mg/kg, respectively: Table S2). The contrast in Zn concentration was obtained by supplementing zinc oxide (ZnO) to the control diet. From d 23 till 35, the piglets in both treatment groups received the same starter piglet diet with a regular Zn concentration (analysed 100 mg Zn/kg). This experimental design is schematically presented in Fig. 1A.

#### Production performance

The weight of the piglets was registered on d −1, 14, 23 and 35 post weaning and the feed intake per pen was registered over the periods of d 0–14, 14–23 and 23–35. The plasma concentration of Zn was measured in the piglets on d 14, 23 and 35. The results for body weight gain, daily feed intake (per pen), feed conversion ratio, and plasma Zn concentrations were statistically analysed by analysis of variance (ANOVA) [35].

The data were analysed using “dietary treatment” as main experimental factor in the statistical model. An effect of treatment was significant when the probability of having no effect was less than 5% (*P* < 0.05). Differences between treatment means were evaluated by using the *least significant difference* test.

#### Blood and sample collection

On different time-points, i.e., d 14, 21, 35 post weaning, piglets for intestinal sample collection were sedated by administrating pentobarbital (24 mg/kg Euthasol 20%) and thereafter were sacrificed by bleeding. Blood samples were collected at d 14, 21, 35 post weaning, in mineral-free tubes for the determination of the concentration of zinc in the blood plasma. For both jejunum and ileum, digesta from the middle to distal part, with a length of approximately 10 cm, was collected for microbiome analysis, by gently stripping the gut segment into a plastic container, and immediately snap-frozen in liquid nitrogen and subsequently stored at −80 °C until further analysis. For the gene expression analysis, mucosal scrapings of mid-jejunum and mid-ileum were acquired.

#### 16S rRNA amplicon sequencing

##### DNA Extraction

For the microbial DNA extraction, the protocol as described by de Greeff et al*.* [36] was used. After that for bacterial amplicon library preparation a PCR was performed, i.e., 20 cycles, to amplify the 16S rRNA gene. For this we targeted the hypervariable region V3 fragment using the forward primer V3_F (CCTACGGGAGGCAGCAG) and the reverse primer V3_R (ATTACCGCGGCTGCTGG) [37]. The amplicons were checked on agarose gel for their quality, each sample was bar-coded with Illumina adapters and sequenced using paired-end sequencing, 2 × 150 bp technology on a MiSeq sequencer (Illumina, San Diego, CA, USA) at a sequencing depth in the range of 471 K to 1.06 M read-pairs per sample (median 802,810 read-pairs per sample).

##### Pre-processing and statistical analysis

The phyloseq object creation and statistical analyses were performed in R 3.6.1, the associated rds object (Zn_study1.rds) is online available (10.5281/zenodo.7736253). Briefly, the amplicon sequences were quality filtered, primer-trimmed, error-corrected, dereplicated, chimera-checked, and overlapping R1 and R2 sequences were merged using the dada2 package v.1.4.0 [38]. By using the standard parameters except for *TruncLength* = 140,100 and minOverlap = 10, and reads were classified with the SILVA v.132 classifier [39]. The statistical analyses of the taxonomic distributions were performed with the *phyloseq*-v1.34.0 [40], *microbiome*-v1.12.0 [41] and *vegan*-v2.5-7 [42] packages. Prior to analyses, the data were rarefied to 436,997 per sample (*rarefy_even_depth,* set.seed = 111) to allow diversity comparisons and the final dataset contained 5,211 taxa.

Alpha-diversity measures were performed by *estimate_richness* and *evenness* functions of the phyloseq package, for the observed species, Shannon index and Pielou's evenness. Beta-diversity measures were performed by the *ordinate* function (PCoA; Bray–Curtis dissimilarity) of the phyloseq package, followed by the functions *adonis* (phyloseq), *adonis.pair* (EcolUtils 0.1), and *betadisper* (phyloseq) at default settings.

Further statistical testing for compositional differences was performed by *DESeq2* (v1.30.1). The dataset of study 1 was first filtered on detection, i.e., count > 228, and secondly the bacterial groups should be prevalent in at least 5% of the samples. Subsequently, the genera significantly differing in abundance were filtered on their average relative contribution, i.e., above 0.01%, because we assume that these bacterial groups have a larger impact on the ecosystem and functionality.

#### Host transcriptomics

##### RNA extraction tissue

Total RNA was extracted from 50 to 100 mg tissue mucosal scrapings of mid-jejunum and mid-ileum. The jejunum and ileum samples were homogenised using the TisuPrep Homogenizer Omni TP TH220P in TRizol reagent (Life Technologies, Bleiswijk, the Netherlands) as recommended by the manufacturer with minor modifications. The homogenised tissue samples were dissolved in 5 mL of TRizol reagent. After centrifugation the supernatant was transferred to a fresh tube. Subsequently a phase separation with chloroform was performed as described by the manufacturer Life Technologies. The RNA was precipitated and dissolved and quantified by absorbance measurements at 260 nm. Quality Control was performed by Agilent Bioanalyser (Amstelveen, the Netherlands).

##### Labelling, hybridization, scanning and feature extraction

Labelling of RNA was done as recommended by Agilent Technologies using the One-Color Microarray-Based Gene Expression Analysis Low Input Quick Amp Labelling. The input was 10 ng of total RNA and 600 ng of labelled cRNA was used on the eight-pack array. Hybridization was performed as described in the One-Color Microarray-Based Gene Expression Analysis Low Input Quick Amp Labelling protocol from Agilent in the hybridization oven (G2545A hybridization Oven Agilent Technologies). The hybridization temperature was 65 °C with rotation speed 10 r/min for 17 h. After 17 h the arrays were washed as described in the One-Color Microarray-Based Gene Expression Analysis Low Input Quick Amp Labelling protocol from Agilent. The arrays were scanned using the DNA microarray scanner with Surescan high resolution Technology from Agilent Technologies. Agilent Scan Control with resolution of 5 μm, 16 bits and PMT of 100%. Feature extraction was performed using protocol 10.7.3.1 (v10.7) for one colour gene expression. Geo accession number GSE94370 (https://www.ncbi.nlm.nih.gov/geo/query/acc.cgi?acc=GSE94370).

##### Data analysis

The data were analysed by using R (v4.0.2) by executing different packages, including LIMMA and arrayQualityMetrics. The data were read and background corrected (method = "normexp" and offset = 1) with functions from the R package LIMMA [1] from Bioconductor [2]. Quantile normalisation of the data was performed between arrays. The duplicate probes mapping to the same gene were averaged (‘avereps’) and subsequently the lower percentile of probes was removed from the dataset in a three-step procedure, 1) get the highest of the dark spots to get a base value, 2) multiply by 1.1 and 3) the gene/probe must be expressed in each of the samples in an experimental condition (e.g., in ileum d 14 control). We removed the following pigs due to poor quality during QC and/or being an outlier, for jejunum pigs 7745 (d 14 group 1_LLL), 7768 and 7762 (d 23 group 1_LLL), 7872 (d 35 group 1_LLL), and 7779 (d 35 group 1_LHL), whereas for ileum pigs 7767 (d 23 group 1_LLL) and 7774 (d 35 group 1_LHL).

### Study 2

#### Experimental design

The second study was also performed in the experimental facilities of Agrifirm, Laverdonk in Heeswijk-Dinther, the Netherlands (AVD 401002016416). The piglets were of the Tempo × Topigs 20 genotype. At the start of the study, the piglets were divided per pen in a way that the mean body weight was 7.6 ± 0.8 kg. Male (boars) and female piglets were equally distributed over pens. Piglets were weaned at a mean age of 28 ± 1.2 d. The piglets received creep feed during the suckling period. The piglets were housed in floor pens (1.75 m × 3.00 m) with 12 piglets per pen. Litter mates were spread equally over the available pens as far as possible. The piglets were fed ad libitum using a dry feed dispenser. The diets were provided as pellets. The animals had free access to water via an automatic drinking device.

The ingredient and calculated nutrient composition of the low Zn weaner and starter diets are given in Tables S3 and S4. The diets were formulated to be nutritionally adequate using data on the composition and nutritional value of feed ingredients according to CVB [34]. After weaning, the piglets were fed a weaning experimental diet with a low or high Zn concentration during the period of d 0 till d 14. From d 14 till d 23, piglets in the four treatment groups received an experimental starter diet with a low (142 mg Zn/kg) or high (2,790 mg Zn/kg) Zn concentration. The contrast in Zn concentration in the experimental diets was obtained by supplementing zinc oxide (ZnO) to the low Zn diets. From d 23 till 35, the remaining piglets in each of the four treatment groups received the same starter diet with a low Zn concentration. This experimental design is schematically presented in Fig. 1B.

#### Production performance

The same approach as described for study 1 was used for obtaining the relevant data.

#### 16S amplicon sequencing

The same approach as described for study 1 was used, the associated rds object (Zn_study2.rds) is online available (10.5281/zenodo.7736253). Below we show the parameterization and results that were specific for study 2. For the 16S amplicon sequencing data, prior to analyses, data were rarefied to 252,083 per sample (*rarefy_even_depth,* rngseed = 111) to allow diversity comparisons. The final dataset contained 6,437 taxa.

#### Host transcriptomics

The same approach as described for study 1 was taken. We removed data of the following pigs due to poor quality during QC and/or being an outlier, for jejunum pigs 7222 (d 23 group 2_HLL), whereas for ileum pigs 7132 (d 14 group 2_LLL), 7309 (d 23 group 2_LLL) and 7121 (d 23 group 2_LHL).

## Results

### Study 1

#### Production performance and Zn concentration in blood plasma

Body weight and zootechnical performance parameters over time of the piglets per treatment throughout the study are provided in Table S5. Body weight gain (BWG) was significantly higher in 1_LHL between d 14 and 23 post weaning compared to 1_LLL, 440 compared to 400 respectively. Body weight at d 35 and the feed conversion ratio (FCR) over the entire experimental period (d 0–35 post-weaning) did not significantly differ between treatment groups.

On d 14 post-weaning prior to the experimental treatment, there was no difference in Zn concentration in blood between 1_LLL and 1_LHL. On d 23 post-weaning, the Zn concentration in plasma was significantly higher in 1_LHL piglets (*P* < 0.05, Table S6) compared to 1_LLL piglets, respectively 32.5 μmol/mL and 17.3 μmol/mL. On d 35 post-weaning, twelve days after feeding the same low Zn starter diet to both experimental groups, piglets in 1_LLL and 1_LHL still showed significantly different blood Zn concentration, respectively 16.7 μmol/mL and 19.0 μmol/mL, although levels of 1_LHL were already decreasing compared to d 23.

#### 16S amplicon sequencing

In the alpha-diversity measures, we observed significance for the Observed species, i.e., richness, at d 35 in jejunum, where 1_LLL had 692 species compared to 1,165 species in 1_LHL and a trend in ileum, where 1_LLL had 782 species compared to 615 species in 1_LHL (Table 1). To assess beta-diversity of the microbiome, we first performed a principal coordinate analysis (PCoA, Fig. S1). The resulting principal coordinates (PCs), principal component (PC) 1 explained 32.8% and PC2 explained 20.8% of the variability. Subsequently, a permutational multivariate analysis of variance using distance matrices was performed with a full model with three-way interactions. This resulted in statistical significance for the interactions day × tissue and day × treatment, as well as day and tissue. A trend was observed for treatment (Table 2). Further testing was performed by employing pairwise tests. The factor treatment was only significant for d 35 in jejunum.

**Table 1:** Alpha diversity of gut microbiota per treatment group on d 14, 23 and 35 in study 1

Alpha diversity	Treatment	Jejunum			Ileum		
		**14**	**23**	**35**	**14**	**23**	**35**
Observed	1_LLL	833 (± 381)	813 (± 418)	692 (± 238)	532 (± 150)	517 (± 195)	782 (± 196)
	1_LHL		777 (± 321)	1165 (± 248)		655 (± 171)	615 (± 164)
	*P*		**0.7**	**0.02**		**0.13**	**0.09**
Shannon	1_LLL	3.29 (± 0.55)	3.57 (± 1.08)	3.24 (± 0.79)	2.87 (± 0.42)	2.73 (± 0.53)	3.60 (± 0.48)
	1_LHL		3.41 (± 0.91)	3.62 (± 0.44)		3.33 (± 0.63)	3.39 (± 0.28)
	*P*		**0.82**	**0.24**		**0.13**	**0.48**
Pielou’s evenness	1_LLL	0.49 (± 0.06)	0.54 (± 0.12)	0.50 (± 0.10)	0.46 (± 0.05)	0.44 (± 0.07)	0.54 (± 0.06)
	1_LHL		0.51 (± 0.10)	0.51 (± 0.06)		0.51 (± 0.09)	0.53 (± 0.03)
	*P*		**0.82**	**0.48**		**0.13**	**0.94**

**Table 2:** Permutational multivariate analysis of variance of gut microbiota data using distance matrices in study 1

Fixed effects	R^2^	Pr (> F)	Day	Tissue	Treatment	R^2^	*P*^a^	*P*_adj_
Day	0.13	0.001	35	Jejunum	1_LLL vs. 1_LHL	0.31	0.01	0.02
Tissue	0.09	0.001	14 vs. 35	Ileum	1_LLL	0.38	0	0.02
Treatment	0.02	0.08	23 vs. 35	Jejunum	1_LHL	0.25	0.01	0.03
Day × Tissue	0.04	0.02	23 vs. 35	Ileum	1_LLL	0.37	0.01	0.03
Day × Treatment	0.04	0.01	23 vs. 35	Ileum	1_LHL	0.26	0.02	0.04
Tissue × Treatment	0.01	0.31	35	Jejunum vs. Ileum	1_LLL	0.4	0	0.02
Day × Tissue × Treatment	0.01	0.88	35	Jejunum vs. Ileum	1_LHL	0.37	0.01	0.02
Residuals	0.66							
**Total**	**1**							

^a^permutational MANOVA

Significance was observed for d 14 versus 35 in treatment 1_LLL in ileum, whereas d 23 versus 35 significance was observed in the 1_LHL treatment and for ileum in both treatments. For tissue only at d 35 significant differences were observed for both treatments. The permutation test for homogeneity of multivariate dispersions showed only one significant finding for d 23 vs. 35 in ileum treatment 1_LHL (permuted *P*-value = 0.01). The next analysis was to visualize the compositional differences on different taxa levels, i.e., phyla (Fig. S2A) and (top 10 abundant) genera (Fig. S2B). When testing for significant differences at genera level between the treatments (1_LLL vs. 1_LHL) per tissue (jejunum and ileum) for each time-point separately, we filtered the rarefied data to be detected with a count of 4,370 (is 0.01%) or more and a prevalence of 5%. This will ensure that we remove taxa that have a small mean and trivially large coefficient of variance. For jejunum, 25 genera were included for further analysis and one genus for d 23 was significant, whereas for d 35, ten genera were significant (Table 3). For ileum, 20 genera passed the filtering, six genera for d 23 were significant and none for d 35 (Table 3).

**Table 3:** Significant genera (relative abundance, *P*_adj_ < 0.05) when comparing treatment 1_LHL vs. 1_LLL in jejunum and ileum at d 23 and 35 in study 1

Tissue	Day	Genus	ARC 1_LLL	ARC 1_LHL	log_2_ fold change	lfcSE^a^	*P*	*P*_adj_^b^
Jejunum	23	*Anaerococcus*	0.009	0.166	4.86	0.99	9.79E-07	2.35E-05
	35	Clostridiaceae^c^	1.066	4.378	1.81	0.73	1.30E-02	3.46E-02
		Peptostreptococcaceae^c^	0.696	3.611	2.2	0.64	6.16E-04	2.46E-03
		*Anaerococcus*	0.024	1.003	4.37	0.98	9.09E-06	7.27E-05
		*Corynebacterium*	3.067	0.936	–2.73	0.69	8.26E-05	3.97E-04
		*Lactobacillus*	68.858	41.929	–2.22	0.85	9.05E-03	2.71E-02
		*Prevotella*	0.014	0.539	4.5	1.1	4.66E-05	2.80E-04
		*Turicibacter*	1.018	23.366	3.99	0.87	4.99E-06	6.91E-05
		*Veillonella*	0.231	0.123	–2.69	1.13	1.75E-02	4.20E-02
		*Weissella*	3.928	0.228	–5.58	1.23	5.76E-06	6.91E-05
		Chloroplast^c^	0.170	1.525	2.59	0.99	8.93E-03	2.71E-02
Ileum	23	Pasteurellaceae^c^	0.095	0.529	3.47	1.36	1.08E-02	4.98E-02
		*Actinobacillus*	0.408	6.518	3.78	1.26	2.63E-03	1.66E-02
		*Corynebacterium*	0.458	0.836	1.98	0.82	1.57E-02	4.98E-02
		*Helicobacter*	0.053	0.107	–3.32	1.37	1.52E-02	4.98E-02
		*Staphylococcus*	0.295	2.710	3.35	0.81	3.45E-05	6.55E-04
		*Veillonella*	2.695	1.169	–3.59	1.15	1.84E-03	1.66E-02
	35	-			-	-	-	-

^a^log fold change standard error

^b^Benjamini-Hochberg (BH)

^c^This ASV could only be annotated to family level

#### Host transcriptomics

First, a principal component analysis (PCA) was performed, to explore the whole genome expression data in both jejunum and ileum, and the effects of different time-points, as well as the dietary Zn treatments. This resulted in a clear distinction between samples of jejunal and ileal tissue (Fig. S3), whereas the effects of time of sampling and dietary treatment were less clear. Because there was a clear distinction between tissues, we have made separate analysis for each tissue, i.e., jejunum (Fig. S4A) and ileum (Fig. S4B). Where for jejunum PC1 explained 11.2% of the variance and PC2 10.2%, for ileum PC1 explained 12.5% and PC2 9.8%. In both tissues, i.e., jejunum and ileum, the day effect was observed in the gene expression patterns, mainly discriminating on PC2, whereas the treatment effect was mainly observed at d 23.

Differences in expression of specific genes as affected by dietary treatment (1_LLL vs. 1_LHL) were explored on d 23 and 35 for both jejunum and ileum. When comparing 1_LLL vs. 1_LHL at d 23, two significantly expressed genes were observed in jejunum tissue and three genes in ileum tissue (Table 4). At d 35, no significant differences in gene expression in intestinal tissues were observed.

**Table 4:** Significantly up- or down-regulated genes in intestinal tissue of piglets on d 23 in study 1^a^

Tissue	Gene	logFC	*P*	*P*_adj_^b^
Jejunum	*C3*	3.07	1.44E-09	5.11E-06
	*MT1A*	4.20	2.12E-08	3.75E-05
Ileum	*C3*	2.83	2.13E-09	7.54E-06
	*MT1A*	3.98	1.68E-08	2.98E-05
	*RENBP*	–1.81	1.06E-05	1.26E-02

^a^*P*_adj_ < 0.01 and logFC < –1.5 or > 1.5

^b^Benjamini–Hochberg corrected

### Study 2

#### Production performance and Zn concentration in blood plasma

Body weight and production performance over time of the piglets per treatment are provided in Supplementary Table S7. Over d 0–14, BWG and feed intake (FI) were significantly higher in the 2_HLL and 2_HHL treatments compared to the 2_LLL and 2_LHL treatments (*P* < 0.05; Table S7). For BWG, this was 262 g/d for 2_HLL and 2_HHL treatments and 221 g/d for 2_LLL and 2_LHL, and for feed intake (FI) this was 344 g/d and 297 g/d, respectively. The FCR did not significantly differ among treatments. Over the periods of d 14–23, 23–35 and over the complete experimental period (d 0–35) (Table S7) BWG, FI, and FCR did not differ between treatments. Also, the body weight at d 35 was not different between treatment groups.

On d 14 post-weaning, there was a significant difference in Zn concentration in blood between 2_LLL or 2_LHL and 2_HLL or 2_HHL (Table 5), 10.3 μmol/mL and 22.8 μmol/mL, respectively. On d 23 post-weaning, the Zn concentration in plasma was significantly higher in 2_LHL piglets (23.1 μmol/mL) compared to 2_LLL (16.9 μmol/mL) and 2_HLL (17.6 μmol/mL), but significantly lower compared to 2_HHL (27.2 μmol/mL) (Table S8). The 2_HHL showed a significant increase compared to all other treatments (Table S8). On d 35 post-weaning, after 12 days of returning to low dietary zinc levels, the plasma Zn concentration did not significantly differ between treatments.

**Table 5:** Alpha diversity of gut microbiota per treatment group on d 14 and 23 in study 2

**Alpha diversity**	Treatment	**d 14**		Treatment	**d 23**	
		**Jejunum**	**Ileum**		**Jejunum**	**Ileum**
Observed species mean (± sd)	2_LLL + 2_LHL	736 (± 466)	431 (± 230)	LLL	660 (± 352)	694 (± 451)
	2_HLL + 2_HHL	689 (± 169)	533 (± 96)	HLL	840 (± 237)	335 (± 120)
				LHL	559 (± 241)	358 (± 119)
				HHL	666 (± 332)	448 (± 129)
	***P***^**a**^	0.96	0.49	***P***^**b**^	0.41	0.11
Shannon mean (± sd)	2_LLL + 2_LHL	3.30 (± 1.01)	3.01 (± 0.25)	LLL	3.21 (± 1.04)	3.53 (± 0.91)
	2_HLL + 2_HHL	3.76 (± 0.68)	3.59 (± 0.28)	HLL	3.46 (± 0.86)	3.11 (± 0.66)
				LHL	3.26 (± 0.79)	3.17 (± 0.57)
				HHL	3.48 (± 1.07)	3.45 (± 0.47)
	***P***^**a**^	0.33	**0.003**	***P***^**b**^	0.97	0.31
Pielou's evenness mean (± sd)	2_LLL + 2_LHL	0.51 (± 0.10)	0.51 (± 0.05)	LLL	0.50 (± 0.13)	0.55 (± 0.10)
	2_HLL + 2_HHL	0.58 (± 0.09)	0.57 (± 0.04)	HLL	0.51 (± 0.11)	0.54 (± 0.09)
				LHL	0.52 (± 0.09)	0.54 (± 0.07)
				HHL	0.54 (± 0.12)	0.57 (± 0.06)
	***P***^**a**^	0.33	**0.02**	***P***^**b**^	0.98	0.80

^a^Wilcoxon signed-rank test

^b^Kruskal-Wallis

#### 16S amplicon sequencing

Alpha-diversity measures showed significant differences for treatment 2_HLL and 2_HHL compared to treatment 2_LLL and 2_LHL in ileum digesta on d 14 for the Shannon index and Pielou’s evenness (Table 5). For the Shannon index, the 2_HLL and 2_HHL was 3.59 compared to 3.01 for treatment 2_LLL and 2_LHL, whereas for Pielou’s evenness this was 0.57 and 0.51, respectively. To assess beta-diversity of the microbiome, we first performed a PCoA (Fig. S5). The resulting principal coordinates (PCs) explained 29.2% for PC1 and 14.0% for PC2. Subsequently, a permutational multivariate analysis of variance using distance matrices, when we ran a full model with three-way interactions, this resulted in significant differences for the interaction day × treatment, as well as day and tissue, and a trend was observed for treatment (Table 6). Further testing was performed by employing pairwise tests (Table 6), for treatment only d 14 in jejunum was significant. For both jejunum and ileum, a significant effect was observed for d 14 versus 23 in treatment 2_HLL. For tissue significant differences were observed for d 14 in treatment 2_LLL and 2_HLL and for d 23 only in 2_HLL. The permutation test for homogeneity of multivariate dispersions was not statistical different.

**Table 6:** Results of permutational multivariate ANOVA and subsequent pair-wise testing of gut microbiota of study 2

Fixed effects	R^2^	Pr (> F)	Day	Tissue	Treatment	R^2^	*P*^a^	*P*_adj_
Day	0.03	0.007	14	Jejunum	2_HLL vs. 2_LLL	0.17	0.01	0.03
Tissue	0.15	0.001	14 vs. 23	Jejunum	2_HLL	0.21	0	0.01
Treatment	0.04	0.05	14 vs. 23	Ileum	2_HLL	0.17	0.02	0.03
Day × Tissue	0.01	0.14	14	Jejunum vs. Ileum	2_LLL	0.17	0.02	0.03
Day × Treatment	0.02	0.02	14	Jejunum vs. Ileum	2_HLL	0.21	0.01	0.02
Tissue × Treatment	0.02	0.73	23	Jejunum vs. Ileum	2_HLL	0.39	0	0.01
Day × Tissue × Treatment	0.01	0.20						
Residuals	0.71							
**Total**	**1**							

^a^Permutational multivariate ANOVA

The next analysis was to visualize the compositional differences on different taxa levels, i.e., phyla (Fig. S6A) and (top 10 abundant) genera (Fig. S6B). When testing for significant differences at the genera level between the treatments (1_LLL vs. 1_LHL) per tissue (jejunum and ileum) for each time-point separately, we filtered the rarefied data with a count of 2,520 (this is 0.01%) or more and a prevalence of 5%. This will ensure that we exclude taxa that have a small mean and trivially large coefficient of variance. For jejunum digesta 25 bacterial genera were used for further analysis. When comparing treatments, i.e., 2_HLL and 2_HHL to 2_LHL and 2_LLL, abundance of three genera of bacteria were observed to be different, whereas for ileum digesta the occurrence of two genera were different (Table 7). For d 23, six treatments were compared, i.e., 2_HHL to 2_LLL, 2_HHL to 2_HLL, 2_HLL to 2_LLL, 2_HHL to 2_LHL, 2_LHL to 2_LLL, and 2_LHL to 2_HLL. For jejunum samples the presence of in total 14 genera were found different and for ileum samples two genera (Table 8).

**Table 7:** Significant genera (relative abundance, *P*_adj_ < 0.05) when comparing treatments in jejunum and ileum at d 14 in study 2

Tissue	Day	Treatment	Genus	2_HLL + 2_HHL	2_LHL + 2_LLL	log_2_ fold change^a^	lfcSE^b^	*P*	*P*_adj_^c^
Jejunum	14	2_HLL + 2_HHL vs. 2_LHL + 2_LLL	*Escherichia*/*Shigella*	1.492	3.996	−4.03	1.03	8.78E-05	1.05E-03
Jejunum	14	2_HLL + 2_HHL vs. 2_LHL + 2_LLL	*Moraxella*	0.834	0.025	4.35	0.8	5.97E-08	1.43E-06
Jejunum	14	2_HLL + 2_HHL vs. 2_LHL + 2_LLL	*Phascolarctobacterium*	0.105	0.377	−2.82	0.83	6.21E-04	4.97E-03
Ileum	14	2_HLL + 2_HHL vs. 2_LHL + 2_LLL	*Sarcina*	0.100	1.107	−6.18	1.27	1.23E-06	2.35E-05
Ileum	14	2_HLL + 2_HHL vs. 2_LHL + 2_LLL	Chloroplast^d^	4.307	0.159	3.08	0.84	2.32E-04	2.20E-03

^a^log fold change is based on count data

^b^log fold change standard error

^c^Benjamini-Hochberg (BH)

^d^This ASV could only be annotated to family or order level

**Table 8:** Significant genera (relative abundance, *P*_adj_ < 0.05) when comparing treatments in jejunum and ileum at d 23 in study 2

**Tissue**	**Day**	**Treatment**	**Genus**	2_HHL	2_HLL	2_LHL	2_LLL	log_2_ fold change^**a**^	**lfcSE**^**b**^	***P***	***P***_**adj**_^**c**^
Jejunum	23	2_HHL vs. 2_LLL	*Lactococcus*	**0.19**	0.11	0.32	**0.06**	2.48	0.75	9.12E-04	5.47E-03
Jejunum	23	2_HHL vs. 2_LLL	*Staphylococcus*	**1.10**	0.77	1.62	**0.19**	2.73	0.67	5.25E-05	6.30E-04
Jejunum	23	2_HHL vs. 2_LLL	*Veillonella*	**17.72**	8.47	12.39	**5.40**	2.78	0.81	5.63E-04	4.50E-03
Jejunum	23	2_HHL vs. 2_LLL	*Weissella*	**2.89**	0.31	1.10	**0.08**	5.66	0.97	5.68E-09	1.36E-07
Jejunum	23	2_HLL vs. 2_LLL									
Jejunum	23	2_HHL vs. 2_HLL	Mitochondria^d^	**0.94**	**0.43**	0.36	1.13	1.93	0.58	9.38E-04	8.65E-03
Jejunum	23	2_HHL vs. 2_HLL	*Veillonella*	**17.72**	**8.47**	12.39	5.40	2.55	0.78	1.08E-03	8.65E-03
Jejunum	23	2_HHL vs. 2_HLL	*Weissella*	**2.89**	**0.31**	1.10	0.08	4.14	0.94	9.75E-06	2.34E-04
Jejunum	23	2_HHL vs. 2_LHL									
Jejunum	23	2_LHL vs. 2_LLL	*Lactococcus*	0.19	0.11	**0.32**	**0.06**	2.71	0.75	2.96E-04	2.37E-03
Jejunum	23	2_LHL vs. 2_LLL	*Moraxella*	0.20	0.09	**0.30**	**0.05**	2.77	0.86	1.22E-03	5.86E-03
Jejunum	23	2_LHL vs. 2_LLL	*Staphylococcus*	1.10	0.77	**1.62**	**0.19**	3.06	0.67	5.79E-06	1.39E-04
Jejunum	23	2_LHL vs. 2_LLL	*Stenotrophomonas*	0.54	0.32	**1.39**	**0.15**	2.89	0.83	5.31E-04	3.18E-03
Jejunum	23	2_LHL vs. 2_LLL	*Weissella*	2.89	0.31	**1.10**	**0.08**	4.24	0.97	1.29E-05	1.54E-04
Jejunum	23	2_LHL vs. 2_HLL	Lachnospiraceae^d^	0.02	**0.03**	**0.01**	0.05	-2.91	0.75	1.08E-04	2.59E-03
Jejunum	23	2_LHL vs. 2_HLL	*Blautia*	0.36	**0.74**	**0.09**	0.16	-2.66	0.79	8.03E-04	9.64E-03
Ileum	23	2_HHL vs. 2_LLL	*Veillonella*	11.33	1.90	1.66	1.40	3.59	0.95	1.55E-04	2.95E-03
Ileum	23	2_HHL vs. 2_HLL									
Ileum	23	2_HLL vs. 2_LLL									
Ileum	23	2_HHL vs. 2_LHL	*Veillonella*	11.33	1.90	1.66	1.40	3.42	0.95	3.21E-04	6.11E-03
Ileum	23	2_LHL vs. 2_LLL									
Ileum	23	2_LHL vs. 2_HLL									

^a^log fold change is based on count data

^b^log fold change standard error

^c^Benjamini-Hochberg (BH)

^d^This ASV could only be annotated to family or order level

#### Host transcriptomics

First, a PCA was performed, to explore the whole genome expression data in both jejunum and ileum, and the effects of different time-points, as well as the dietary zinc treatments. This resulted in a clear distinction between samples of jejunal and ileal tissue (Fig. S7), whereas the effects of time of sampling and dietary treatment were less clear. Because there was a clear distinction between tissues, we have made separate analysis for each tissue, i.e., jejunum (Fig. 8A) and ileum (Fig. 8B). Where for jejunum PC1 explained 10.1% of the variance and PC2 7.5%, for ileum PC1 explained 11.1% and PC2 8.6%. In both tissues a day effect was observed, mainly discriminating on PC2, whereas the treatment effect was mainly observed on d 14 (2_LLL and 2_LHL vs. 2_HLL and 2_HHL). Differences in expression of specific genes as affected by dietary treatment were explored on d 14 and 23 in both jejunum and ileum tissue. When comparing 2_LLL and 2_LHL to 2_HLL and 2_HHL at d 14, 23 significantly expressed genes were observed for jejunum and 11 genes for ileum (Table 9). At d 23 (Table 10) a lower number of differentially expressed genes were observed, only one gene for the comparisons 2_HHL to 2_LLL and 2_LHL to 2_HLL in jejunum, as well as for the comparison 2_HHL to 2_LLL in ileum. In addition, the comparison 2_HHL to 2_HLL yielded eight genes that were expressed differentially.

**Table 9:** Differentially expressed genes in intestinal mucosa on d 14 in study 2^a^

Tissue	Gene	logFC^b^	*P*	*P*_adj_^c^
Jejunum	*MT1A*	5.30	1.74E-10	1.17E-06
	*C3*	2.43	3.36E-06	1.87E-03
	*PWWP3B*	1.94	3.01E-04	1.92E-02
	*HOMEZ*	1.91	1.08E-05	3.29E-03
	*CYP2B22*	1.82	2.20E-03	5.86E-02
	*SMAD1*	1.58	6.59E-05	9.00E-03
	*LOC110255328*	−1.53	4.15E-05	6.60E-03
	*SNAI2*	−1.60	2.74E-03	6.52E-02
	*GPX2*	−1.63	1.86E-05	4.28E-03
	*PCPA1*	−1.67	1.37E-05	3.65E-03
	*CDC42BPA*	−1.76	3.95E-04	2.24E-02
	*WAP-1*	−1.79	3.32E-06	1.87E-03
	*IFIT2*	−1.85	2.51E-03	6.30E-02
	*NLRP2*	−2.02	7.04E-04	3.16E-02
	*HGSNAT*	−2.09	3.81E-08	1.27E-04
	*FATE1*	−2.30	9.80E-06	3.12E-03
	*HEXB*	−2.43	2.21E-03	5.86E-02
	*TP53BP1*	−2.44	7.58E-07	8.45E-04
	*TFF1*	−2.60	1.94E-03	5.58E-02
	*BPIL1*	−2.86	3.63E-05	6.10E-03
	*HNRNPH3*	−2.92	3.65E-05	6.10E-03
	*CD59*	−3.06	5.46E-07	7.30E-04
	*REG3G*	−7.02	7.26E-06	2.70E-03
Ileum	*MT1A*	5.18	1.12E-09	7.51E-06
	*HOMEZ*	1.73	9.44E-05	4.51E-02
	*GPX2*	−1.50	1.12E-04	4.99E-02
	*LBP*	−1.56	1.66E-04	5.84E-02
	*LOC110255328*	−1.62	2.96E-05	2.20E-02
	*FATE1*	−2.41	7.56E-06	8.42E-03
	*LOC414409*	−2.47	5.22E-05	3.20E-02
	*HNRNPH3*	−2.95	5.45E-05	3.20E-02
	*RENBP*	−3.08	1.54E-06	2.58E-03
	*CD59*	−3.50	5.01E-08	1.67E-04
	*REG3G*	−6.95	1.61E-05	1.54E-02

^a^*P*_adj_ < 0.01 and logFC < −1.5 or > 1.5

^b^High ZnO : Low ZnO

^c^Benjamini-Hochberg corrected

**Table 10:** Differentially expressed genes in intestinal mucosa on d 23 when comparing treatments in study 2^a^

Tissue	Contrast	Gene	logFC	*P*	*P*_adj_^b^
Jejunum	HHL vs. LLL	*MT1A*	3.90	1.25E-06	2.78E-03
	LHL vs. HLL	*ARPP21*	3.69	3.12E-07	2.09E-03
Ileum	HHL vs. LLL	*MT1A*	3.97	3.30E-06	2.21E-02
	HHL vs. HLL	*C3*	2.00	1.99E-04	7.84E-02
	HHL vs. HLL	*HOMEZ*	1.90	1.28E-05	3.69E-02
	HHL vs. HLL	*IYD*	–1.55	5.86E-05	4.35E-02
	HHL vs. HLL	*FAU*	–1.58	1.65E-05	3.69E-02
	HHL vs. HLL	*LOC110255328*	–1.75	9.87E-06	3.69E-02
	HHL vs. HLL	*CPNE1*	–2.15	9.35E-05	5.68E-02
	HHL vs. HLL	*FATE1*	–2.24	3.52E-05	4.00E-02
	HHL vs. HLL	*RENBP*	–2.51	5.69E-05	4.35E-02

^a^*P*_adj_ < 0.01 and logFC < –1.5 or > 1.5

^b^Benjamini-Hocberg corrected

## Discussion

Various studies already have shown the effects of dietary ZnO supplementation on preventing or decreasing the incidence of post-weaning diarrhoea in post-weaning piglets [9, 14, 20, 43] primarily focusing on the impact on the piglet’s growth performance and on the clinical incidence of diarrhoea. The underlying biological mechanisms, or precise mode-of-action, of ZnO as dietary intervention have not yet been fully elucidated, although links to increased nutrient absorption/digestibility, intestinal morphology [18] and beneficial effects to the immune system and intestinal integrity [19] have been observed. Finding alternative interventions is also of importance, because from June 2022 it has been prohibited to use high concentrations of ZnO in pigs’ diets in the European Union [44–46]. In the present studies, we have used ZnO as a model dietary intervention, rather than as a target intervention to prevent occurrence of post-weaning diarrhoea. Supplementation of ZnO in diets of post-weaning piglets has shown to increase growth performance (feed intake and body weight gain), reduce occurrence of post-weaning diarrhoea, improve nutrient digestibility, modulate of the immune system, to increase activity of digestive enzymes and antibacterial action, improve of intestinal morphology, and increase antioxidant enzyme levels in the small intestine [47]. Many studies used ETEC challenge models to evaluate the effects of ZnO in post-weaning piglets [20, 21]. In contrast, our study offered the possibility to interrogate the influence of ZnO in the digestive tract of piglets in the absence of pathological conditions as caused by e.g., ETEC. The focus was on evaluating the effects on the intestinal microbiome and on responses of gut tissues based on gene expression analysis. ZnO can have direct effects on both the microbiome and gut tissue, but these can also influence each other indirectly through host-microbe interactions.

### Impact of dietary ZnO in the post-weaning period in clinically healthy piglets

The mode-of-action of ZnO is often studied after a challenge with a gut pathogen, i.e., ETEC, or under suboptimal or poor health status. ZnO can have a direct effect on intestinal functionality in pigs, for example via modulation of cytokines produced by the epithelial lining [21]. In addition, to this host-driven regulation, antibacterial activity of ZnO to a broad spectrum of bacteria has been shown in pathogenic challenged pigs [48], as well as on molecular mechanisms in gut tissue related to the antimicrobial activity of ZnO [49]. We found a higher concentration of Zn in the blood plasma of pigs with high dietary ZnO, indicating that zinc absorption in the gut of piglets is dependent on the dietary level of zinc. Generally, Zn absorption in the intestinal tract of piglets is known to be relatively low with a value of about 14% relative to dietary Zn intake [50, 51]. Moreover, the detected Zn concentration i.e., 2.12 µg/mL (32.5 µmol/mL) in the blood plasma of piglets is below the toxicity threshold (> 3 µg/mL) in pigs. We used clinically healthy pigs and showed effects on the microbiota diversity and composition in digesta of the small intestine. We observed a significant difference in the richness at d 35 post-weaning, where the piglets receiving high ZnO showed a higher richness compared to the piglets receiving low ZnO, 1,165 vs. 692 species, respectively. In study 2, however, we observed significant effects on the Shannon index and Pielou’s evenness at d 14 post-weaning when comparing the piglets receiving high versus low ZnO. Where the Shannon index was 3.59 vs. 3.01, and Pielou’s evenness was 0.57 vs. 0.51, for the piglets receiving high versus low ZnO, respectively. These findings indicate a more diverse small intestinal microbiota in the piglets receiving high ZnO, which is often judged as “healthier”, as more bacterial species can potentially act against putative pathogens, a concept known as competitive exclusion [52, 53]. These results are in line with an earlier study that showed an increase in the richness and Shannon index of the gut microbiota when a diet with a high concentration of ZnO (2,500 mg/kg) was fed [54]. Another study also showed a positive effect of high ZnO (2,500 mg/kg) on the stability and diversity of the microbiota (coliforms) two weeks post-weaning [9]. In both study 1 and 2, we observed significant effects on gut microbiota composition after high ZnO supplementation, however when comparing to literature such results are often contradictory. For example studies observed no effect of high ZnO in ileal microbiota [12, 55], whereas other studies observed transient and long lasting effects on the ileal microbiota [20, 56]. These observed contradictory results could be due to the technique used to measure the microbiota composition, for example 16S amplicon sequencing. This technique can only identify the different taxonomical groups of the microbiota, and not their functionality. Moreover, a gut microbiota composed of different microbial species could still have a similar metabolic functionality. For this reason, data based on only the taxonomic composition of microbiomes are often difficult to interpret. Subsequent analyses that focused on the specific bacterial groups, which were increased or reduced in relative abundance between the treatment groups, could be typed as commensals in the mammalian gut, this included *Lactobacillus*, *Anaerococcus*, *Clostridiaceae*, *Peptostreptococcaceae*, *Corynebacterium*, *Turicibacter*, and *Chloroplast.* Also, a decrease in relative abundance of lactic acid producing bacteria (*Lactobacillus* and *Weissella*) and Prevotella was observed in the high ZnO group, these bacterial groups being more commonly found in animals fed plant-rich diets [57]*.* Taken together, these results on the intestinal microbiota showed that a high-level concentration of ZnO in the diet in the post-weaning period shift the small intestinal microbiota composition. As the shift was observed in a non-pathological condition, these results suggest that mainly commensal bacterial genera differed in the gut of piglets when receiving a diet with either a low or high concentration of ZnO.

We observed only a limited number of differentially expressed genes (DEGs) in study 1 in both jejunum and ileum tissue. The higher expression of *C3* in piglets receiving high ZnO could indicate a modulation of the inflammatory state in the small intestinal tissue. Furthermore, the *C3* gene is known to have an antimicrobial activity [58]. In mice it has already been described that genes involved in the complement system respond to different (oral) challenges, i.e., dietary, drug, or immune [59]. Consequently the complement pathway functioning changes by increased expression of genes *C2*, *C3*, *C4*, *CD55*, and factor H, which may be needed as an early defence mechanism against dysbiosis/infection or a dysfunctional barrier [59]. In addition, the gene encoding for *MT1A* belongs to the metallothionein gene family, which has a high content of cysteine residues that can bind various heavy metals, including zinc. We observed higher expression of *MT1A* in piglets receiving high versus low ZnO, which was also observed in a study by Pieper et al. [60]. The former was expected because we observed higher blood plasma levels of Zn in the piglets receiving the ZnO supplemented diet. The gene encoding renin binding protein (*RENBP*) was lower expressed in piglets receiving high ZnO. One of the main functions of renin is to mediate the volume of extracellular fluid and arterial vasoconstriction, i.e., regulating the body’s mean arterial blood pressure. In humans, mRNA for renin has been detected in small intestinal tissue [61]. In pigs, however, data on the expression of this gene in gut tissue are lacking. Taken together, the minor responses in gene expression in small intestinal tissue seems a reflection of the inter-change of commensal bacteria in the gut by dietary ZnO, i.e., a non-pathological modulation of the microbiome composition.

### Impact on intestinal functionality of the timing of the supplementation of dietary ZnO

We have shown some aspects of the mode-of-action at the molecular level by highlighting the direct and indirect effects of high ZnO in the diet on intestinal functionality in clinically healthy pigs. The post-weaning phase, however, is associated with the adaptation of the gut to the intake of solid feed and with different social and environmental stressors imposed which can lead to a “growth check” of piglets [62]. Numerous nutritional and management interventions are described as beneficial for pig health and welfare during the post weaning phase, for example via supplementation of biological active compounds in the sow diet [63], or direct interventions of biological active compounds in piglets [64], such as application of pre- or probiotics via the diet, or application of faecal microbiome transplants [65]. These extrinsic stimuli have an impact on the intestinal functionality, which is also depicted by a change in microbiome composition over time when comparing the early and late post-weaning phase. In the late post-weaning phase, a more diverse microbiome is generally observed [66, 67]. To investigate the impact on intestinal functionality of the timing of a model dietary intervention, here supplementation of ZnO, a second study was performed that contained more treatment groups based on different timings of supplementation. In study 2, ZnO was supplemented immediately post weaning (d 0–14) and/or after this phase (d 14–23), again in clinically healthy pigs. For the small intestinal microbiota, we observed significant changes in the alpha diversity only at d 14 in the ileum, with an increased Shannon and Pielou’s evenness in piglets receiving high versus low ZnO. These results are similar to Pieper et al*.* [54] who observed significantly higher species richness, Shannon diversity, and evenness in the ileum digesta of pigs receiving a high ZnO diet. For the gut microbiota composition, only a significant difference in jejunum at d 14 between piglets receiving high versus low ZnO was observed. This suggests that only minor changes or shifts occur in the small intestinal microbiota after supplementation of ZnO. Interestingly, in study 2 we did not observe similar results in the gut microbiota in the different treatment groups compared to study 1. This might reflect the limitation of the employed sequencing technology in the present study, i.e., amplicon sequencing, which measures the presence of bacterial species rather than their function. Although the observed differences in gut microbiota composition were limited, we did observe 23 DEGs in jejunum and 11 DEGs ileum at d14 between high and low ZnO piglets. More DEGs were observed for the immediately postweaning (d 0–14) supplementation of high ZnO compared to supplementation over d 14–23. When considering the known biological functions of these genes, we again observed increased expression of MT1A for multiple comparisons, i.e., d 14 piglets receiving high versus low ZnO in both jejunum and ileum, as well as d 23 2_HHL vs. 2_LLL in jejunum and 2_HHL vs. 2_LLL in ileum. The increased expression of the *MT1A* gene was expected, because this gene can actively bind zinc in intestinal tissue and is linked to the higher levels of zinc in the blood. Furthermore, we also observed changes in expression of genes involved in immunity. For jejunum the involved genes were *CD59*, *C3*, *IFIT2*, *TP53BP1*, *HEXB*, *HGSNAT*, and *NLRP2*, where only *C3* was increased in expression in the piglets receiving high ZnO and all other showed decreased expression. In ileum tissue the involved genes were *CD59*, *REG3G*, and *LBP*, all showing a decreased expression in the high ZnO group. The gene expression data suggest that activity of inflammatory processes in the small intestine, mainly jejunum, is dampened when feeding a diet with a pharmaceutical level of ZnO. This was also observed in a previous study in pigs, where ZnO supplementation was associated with a decrease of genes involved in inflammation [21]. The dampening in inflammation responses suggests that the challenge by pathogens in the gut in clinically healthy pigs might be lower and consequently impacts the piglet’s resilience in the post-weaning phase. Further, we found that the timing of a ZnO intervention has an impact on intestinal functionality, where immediately post weaning (d 0–14) greater effects were observed compared to d 14–23 post weaning. Better understanding of these dynamics and plasticity of the intestinal development of pigs, in combination with the establishment of the intestinal microbiome and its re-establishment in the immediate post-weaning phase, is important for the health status of pigs. The present work may further aid in the search and application of new feed ingredients and additives targeted to increase and support gut health and thereby growth performance. Such feeding strategies have already been reviewed by Bonetti et al*.* [47]. Our data showed that the timing of a dietary intervention in the post-weaning phase is of importance. The immediate post-weaning period seems the most appropriate window for such interventions.

## Conclusions

We have shown that providing a diet with a high level of ZnO in clinically healthy piglets in the post-weaning phase has multiple effects on intestinal functionality. The alpha diversity of the intestinal microbiome increased and specific genes, such as *CD59* and *REG3G*, in intestinal tissue linked to immunity increased in expression as well. These effects do not seem related to the direct antimicrobial activity of ZnO. Finally, we conclude that ZnO supplementation in the diet as model intervention had the highest impact on intestinal functioning of piglets in the first two weeks post-weaning.

### Supplementary Information

**Additional file 1: Fig. S1. **Principal coordinate analysis using the Bray Curtis dissimilarities of the microbiome in jejnum and ileum of animals in study 1.** Fig. S2. **Compositional data on phylum and genus level averaged over tissue, day, and treatment of study 1.** Fig. S3. **Principal Component Analysis of pig gene expression in both jejunum and ileum together at d 14, 23, and 35 per treatment group (1_LLL and 1_LHL) of study 1.** Fig. S4. **Principal Component Analysis of gene expression in jejunum and ileum tissue at d 14, 23, and 35 per treatment group (1_LLL and 1_LHL).** Fig. S5. **Principal coordinate analysis using the Bray Curtis dissimilarities of the microbiome in jejnum and ileum of animals in study 2.** Fig. S6. **Compositional data on phylum and genus level averaged over tissue, day, and treatment of study 2.** Fig. S7. **Principal component analysis of pig gene expression in both jejunum and ileum together at d 14 and 23 per treatment group (2_LLL, 2_LHL, 2_HLL, and 2_HHL) of study 2.** Fig. S8. **Principal Component Analysis of gene expression in jejunum and ileum tissue at d 14, and 23 per treatment group (2_LLL, 2_LHL, 2_HLL and 2_HHL).** Table S1. **Calculated ingredient and nutrient composition of the weaning and control experimental starter diets (g/kg) of study 1.** Table S2. **Analysed nutrient composition (g/kg dry matter) of the experimental diets provided during d14–35 of study 1.** Table S3. **Calculated ingredient and nutrient composition of the low zinc weaner diet and the low zinc starter diet (g/kg) of study 2.** Table S4. **Analysed nutrient composition (g/kg) of the experimental weaning (d 0–14) and starter diets (d 14–35) of study 2.** Table S5. **Comparison per treatment of body weight (BW) on d −1, 14, 23 and 35, body weight gain (BWG), feed intake and feed conversion ratio (FCR) of the pigs over the periods d 0–14, 14–23, 23–35 and d 0–35 in study 1.** Table S6. **Zinc concentrations1 in blood plasma on d 14, 23 and 35 of animals in study 1.

## Data Availability

The raw microbiota data are available at NCBI under accession PRJNA957270 (Zn study 1) and PRJNA957316 (Zn study 2). In addition, the phyloseq objects (.rds) of the microbiota data for both studies (Zn_study1.rds and Zn_study2.rds) are online and available at https://doi.org/10.5281/zenodo.7736253. Whereas the gene expression data is deposited in the Gene Expression Omnibus (GEO) under accession number GSE94370 (https://www.ncbi.nlm.nih.gov/geo/query/acc.cgi?acc=GSE94370).

## Abbreviations

ANOVA: Analysis of variance
BWG: Body weight gain
DEG: Differentially expressed gene
ETEC: Enterotoxigenic *Escherichia coli*
FCR: Feed conversion ratio
FI: Feed intake
PC: Principal component
PCA: Principal component analysis
PCoA: Principal coordinate analysis
Zn: Zinc
ZnO: Zinc oxide
1_LLL: Study 1, group that received low zinc during all three phases (d 0–35)
1_LHL: Study 1, group that received high zinc during second phase (d 14–23)
2_HLL: Study 2, group that received high zinc during first phase (d 0–14)
2_HHL: Study 2, group that received high zinc during first and second phase (d 0–23)
2_LLL: Study 2, group that received low zinc during all three phases (d 0–35)
2_LHL: Study 2, group that received high zinc during second phase (d 14–23)
