# Supplementary material for: Dietary supplementation of zinc oxide modulates intestinal functionality during the post-weaning period in clinically healthy piglets
Source: J Anim Sci Biotechnol. 2023 Oct 4;14:122. doi: 10.1186/s40104-023-00925-1 (PMC10548679; doi:10.1186/s40104-023-00925-1)
Supplement: Supplementary file 1 — Additional file 1: Fig. S1. Principal coordinate analysis using the Bray Curtis dissimilarities of the microbiome in jejnum and ileum of animals in study 1. Fig. S2. Compositional data on phylum and genus level averaged over tissue, day, and treatment of study 1. Fig. S3. Principal Component Analysis of pig gene expression in both jejunum and ileum together at d 14, 23, and 35 per treatment group (1_LLL and 1_LHL) of study 1. Fig. S4. Principal Component Analysis of gene expression in jejunum and ileum tissue at d 14, 23, and 35 per treatment group (1_LLL and 1_LHL). Fig. S5. Principal coordinate analysis using the Bray Curtis dissimilarities of the microbiome in jejnum and ileum of animals in study 2. Fig. S6. Compositional data on phylum and genus level averaged over tissue, day, and treatment of study 2. Fig. S7. Principal component analysis of pig gene expression in both jejunum and ileum together at d 14 and 23 per treatment group (2_LLL, 2_LHL, 2_HLL, and 2_HHL) of study 2. Fig. S8. Principal Component Analysis of gene expression in jejunum and ileum tissue at d 14, and 23 per treatment group (2_LLL, 2_LHL, 2_HLL and 2_HHL). Table S1. Calculated ingredient and nutrient composition of the weaning and control experimental starter diets (g/kg) of study 1. Table S2. Analysed nutrient composition (g/kg dry matter) of the experimental diets provided during d14–35 of study 1. Table S3. Calculated ingredient and nutrient composition of the low zinc weaner diet and the low zinc starter diet (g/kg) of study 2. Table S4. Analysed nutrient composition (g/kg) of the experimental weaning (d 0–14) and starter diets (d 14–35) of study 2. Table S5. Comparison per treatment of body weight (BW) on d −1, 14, 23 and 35, body weight gain (BWG), feed intake and feed conversion ratio (FCR) of the pigs over the periods d 0–14, 14–23, 23–35 and d 0–35 in study 1. Table S6. Zinc concentrations1 in blood plasma on d 14, 23 and 35 of animals in study 1. [file 40104_2023_925_MOESM1_ESM.docx]

# **Supplementary data**

**
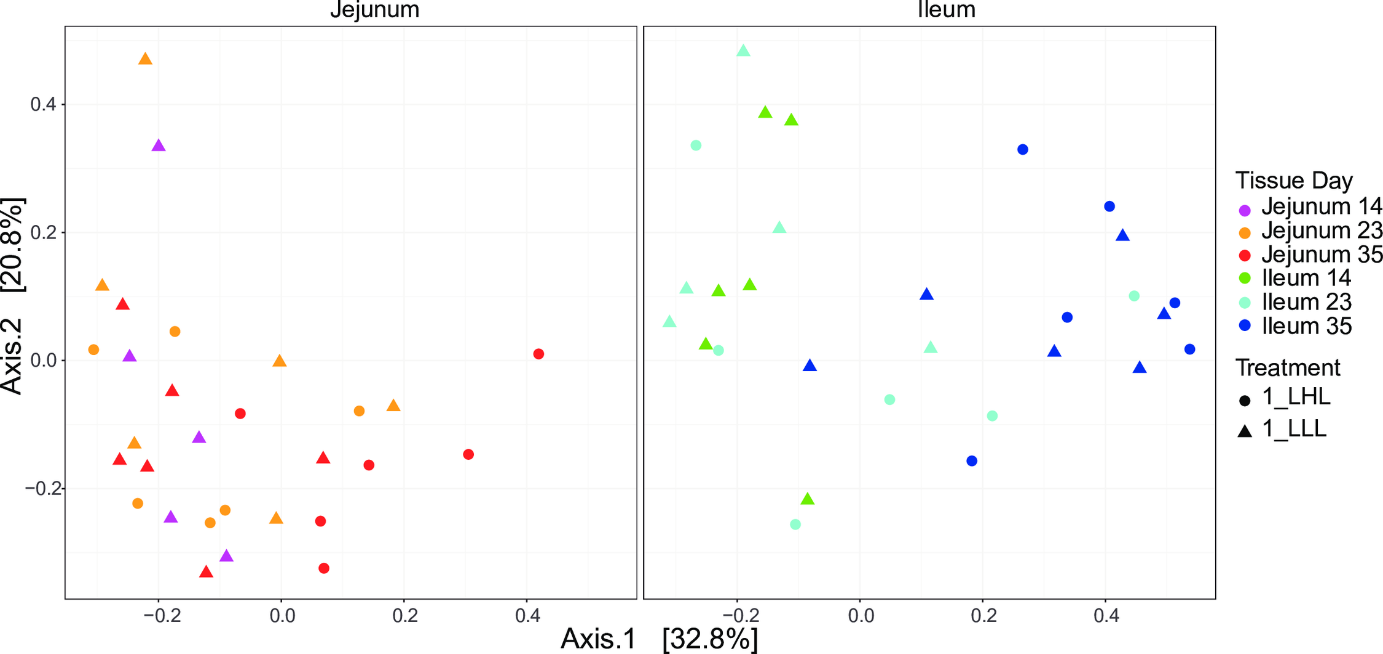
Fig. S1** Principal Coordinate Analysis using the Bray Curtis dissimilarities of the microbiome in jejnum and ileum of animals in study 1. Each symbol represents a sample, where the shape represents the experimental treatment, i.e., circles represent 1_LHL and triangles represent 1_LLL. The color indicates the day for a specific tissue, i.e., for jejunum purple is d 14, orange is d 23, and red is d 35, and for ileum green is d 14, cyan is d 23, and blue is d 35. The first axis explains 32.8% of the variation and the second axis represents 20.8% of the variation.4

**
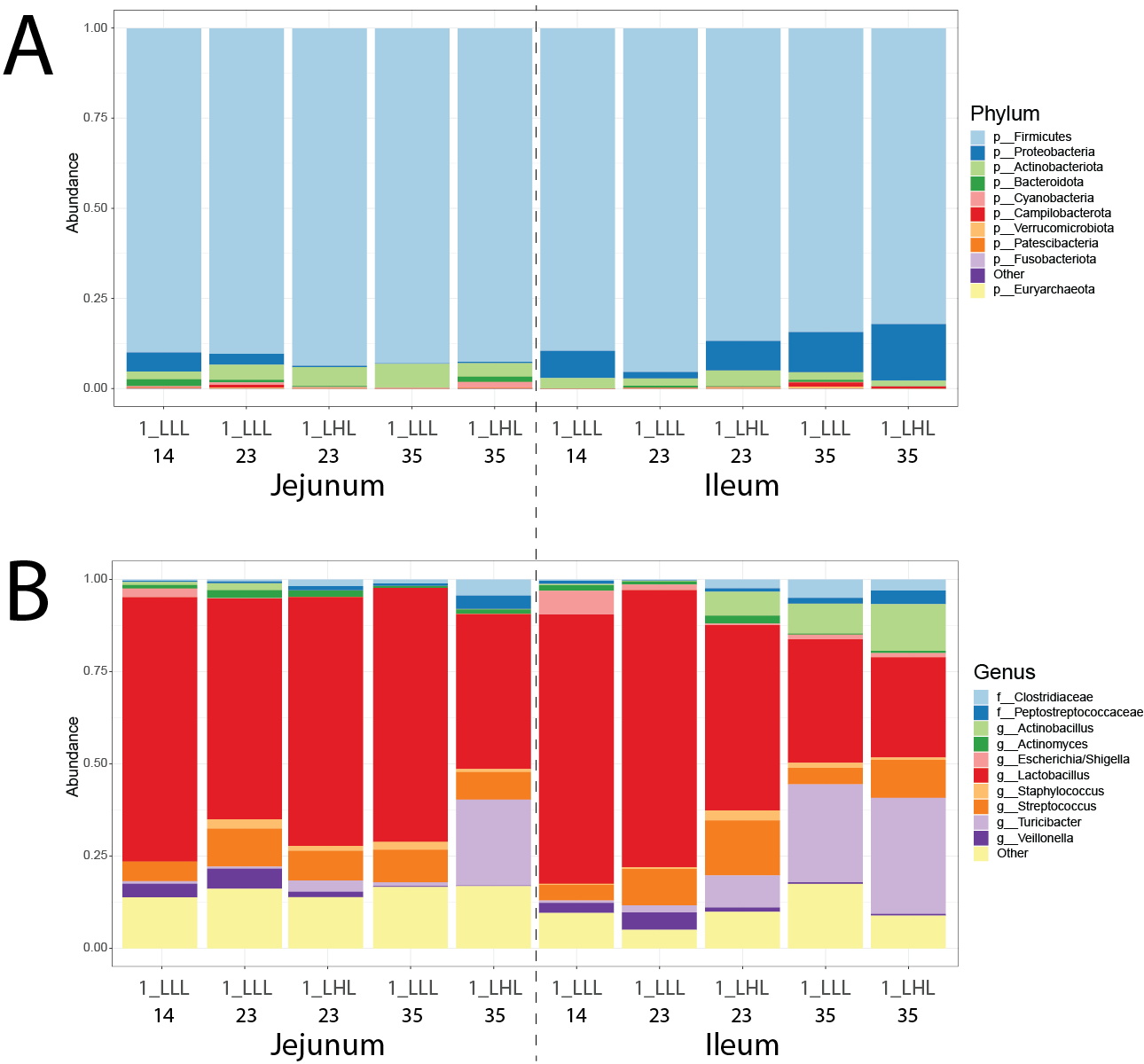
**

**Fig. S2** Compositional data on phylum and genus level averaged over tissue, day, and treatment of study 1. Panel **A** shows the data on phylum level, whereas panel **B** shows the data for genus level. Each color represents a specific phyla or genera as indicated by the respective legends


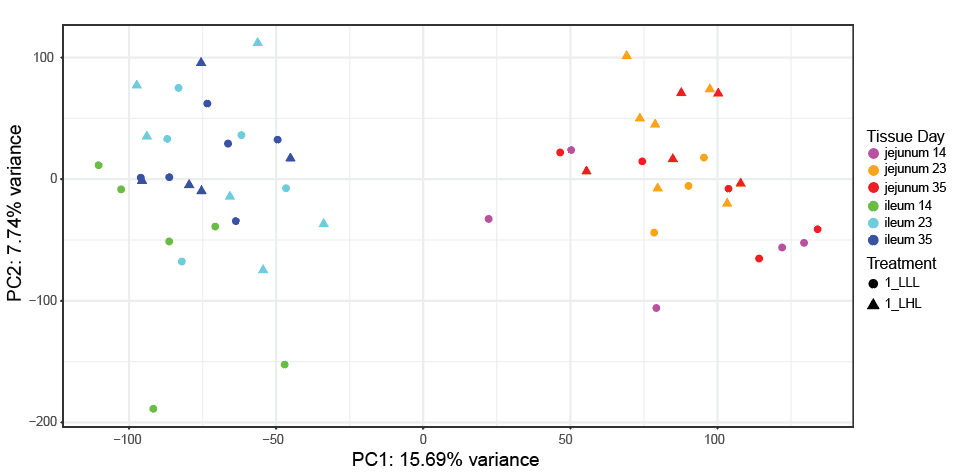
 **Fig. S3** Principal Component Analysis of pig gene expression in both jejunum and ileum together at d 14, 23, and 35 per treatment group (1_LLL and 1_LHL) of study 1. Each colour represents a day; purple is d 14, orange is d 23, and red is d 35 for jejunum and green is d 14, cyan is d 23, and blue is d 35 for ileum. Symbols depict the treatment; circles are treatment 1_LLL and triangles are treatment 1_LHL. Principal Component (PC)1 explains 15.69% of the variance, whereas PC2 explain 7.74% of the variance

**
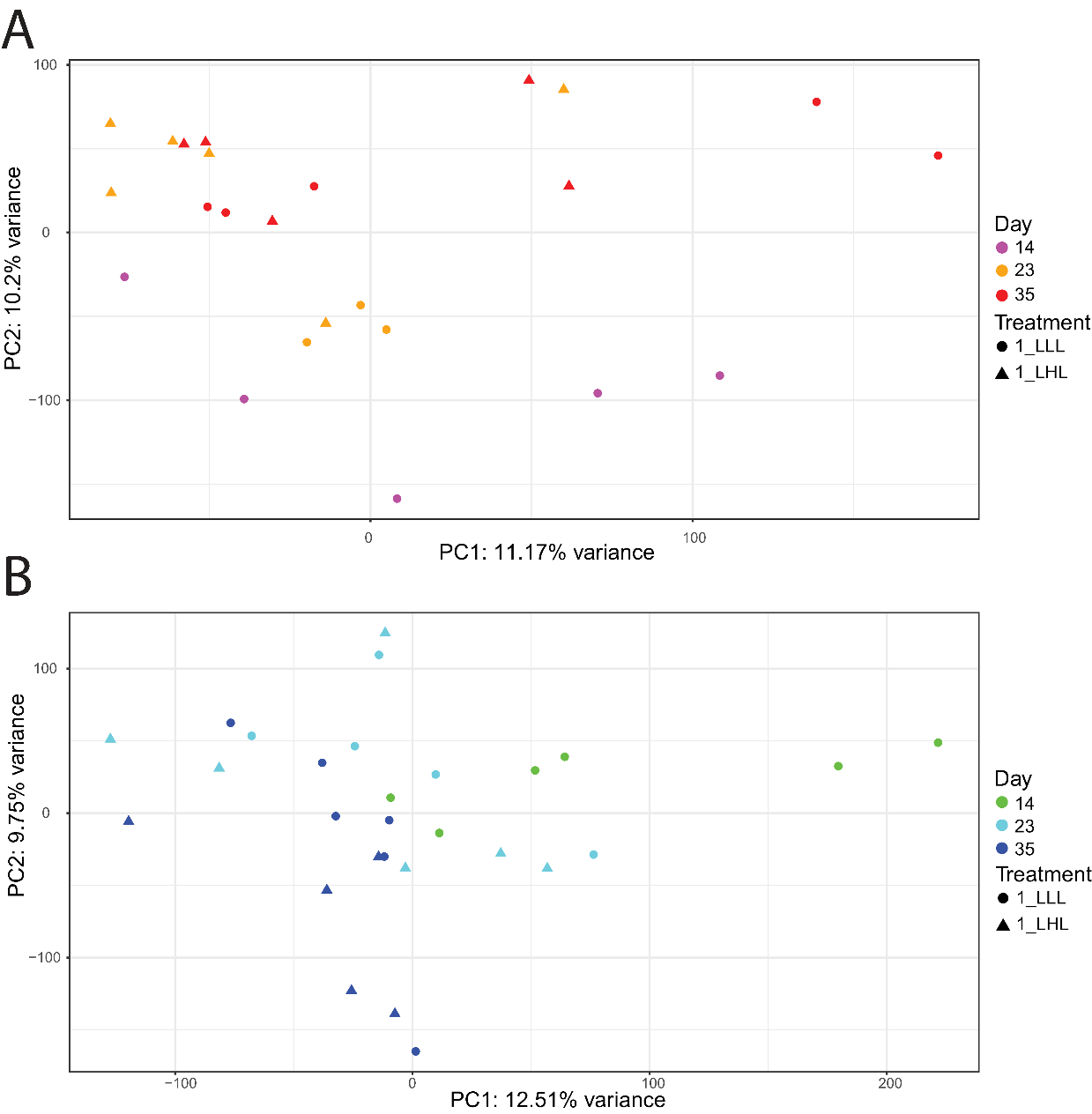
**

**Fig. S4** Principal Component Analysis of gene expression in jejunum and ileum tissue at d 14, 23, and 35 per treatment group (1_LLL and 1_LHL). Panel **A** shows the gene expression in jejunal tissue, where each colour represents a time point in the experiment; purple is d 14, orange is d 23, and red is d 35. Symbols depict the treatment; circles are treatment 1_LLL and triangles are treatment 1_LHL. Principal Component (PC)1 explains 11.2% of the variance, whereas PC2 explain 10.2% of the variance. Panel **B** shows the gene expression in ileum tissue, where each colour represents a time point in the experiment; green is d 14, cyan is d 23, and blue is d 35. Symbols depict the treatment; circles are treatment 1_LLL and triangles are treatment 1_LHL. Principal Component (PC)1 explains 12.5% of the variance, whereas PC2 explain 9.8% of the variance

**
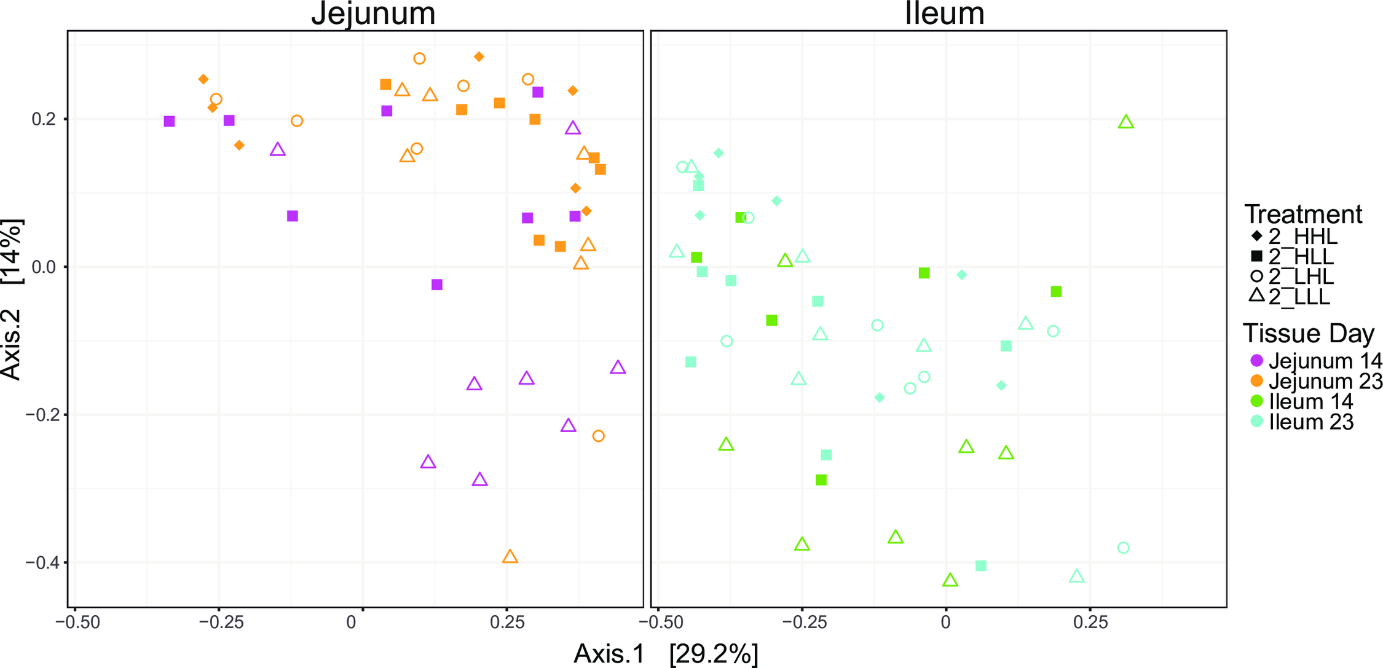
**

**Fig. S5** Principal Coordinate Analysis using the Bray Curtis dissimilarities of the microbiome in jejnum and ileum of animals in study 2. Each symbol represents a sample, where the shape represents the treatment, i.e. diamonds represent 2_HHL, squares represent 2_HLL, open circles represent 2_LHL, and open triangles represent 2_LHL. The color indicates the day for a specific tissue, i.e. for jejunum purple is d 14 and orange is d 23, whereas for ileum green is d 14 and cyan is d 23. The first axis explains 29.2% of the variation and the second axis represents 14.0% of the variation

**
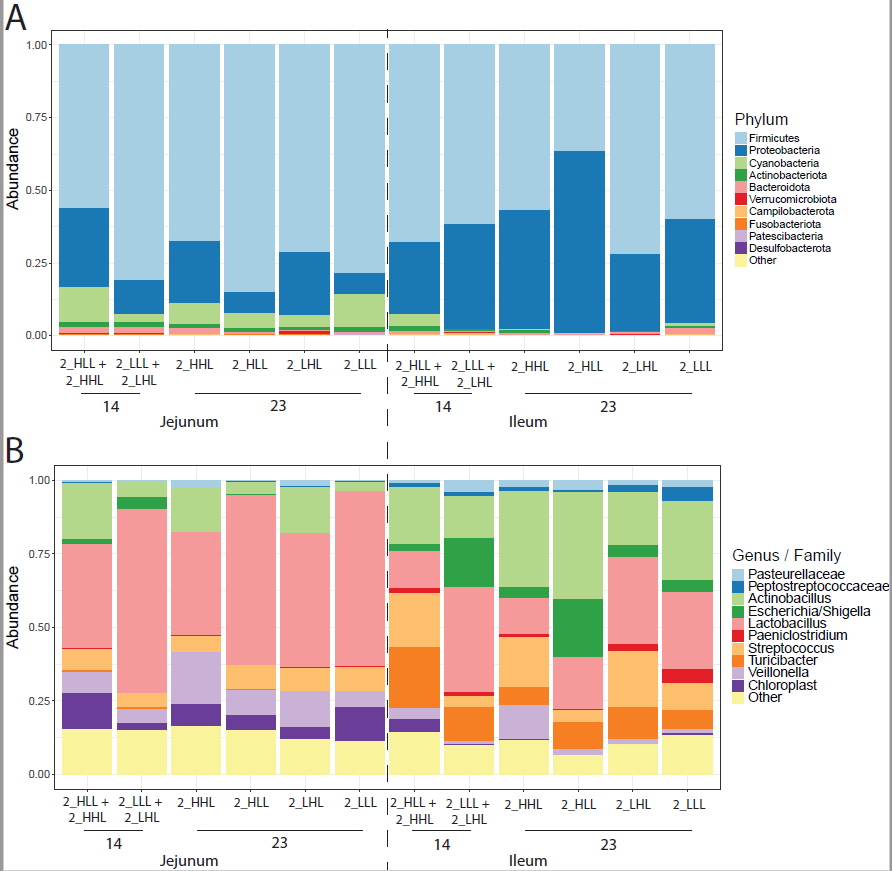
Fig. S6** Compositional data on phylum and genus level averaged over tissue, day, and treatment of study 2. Panel **A** shows the data on phylum level, whereas panel **B** shows the data for genus level. Each color represents a specific phyla or genera as indicated by the respective legends


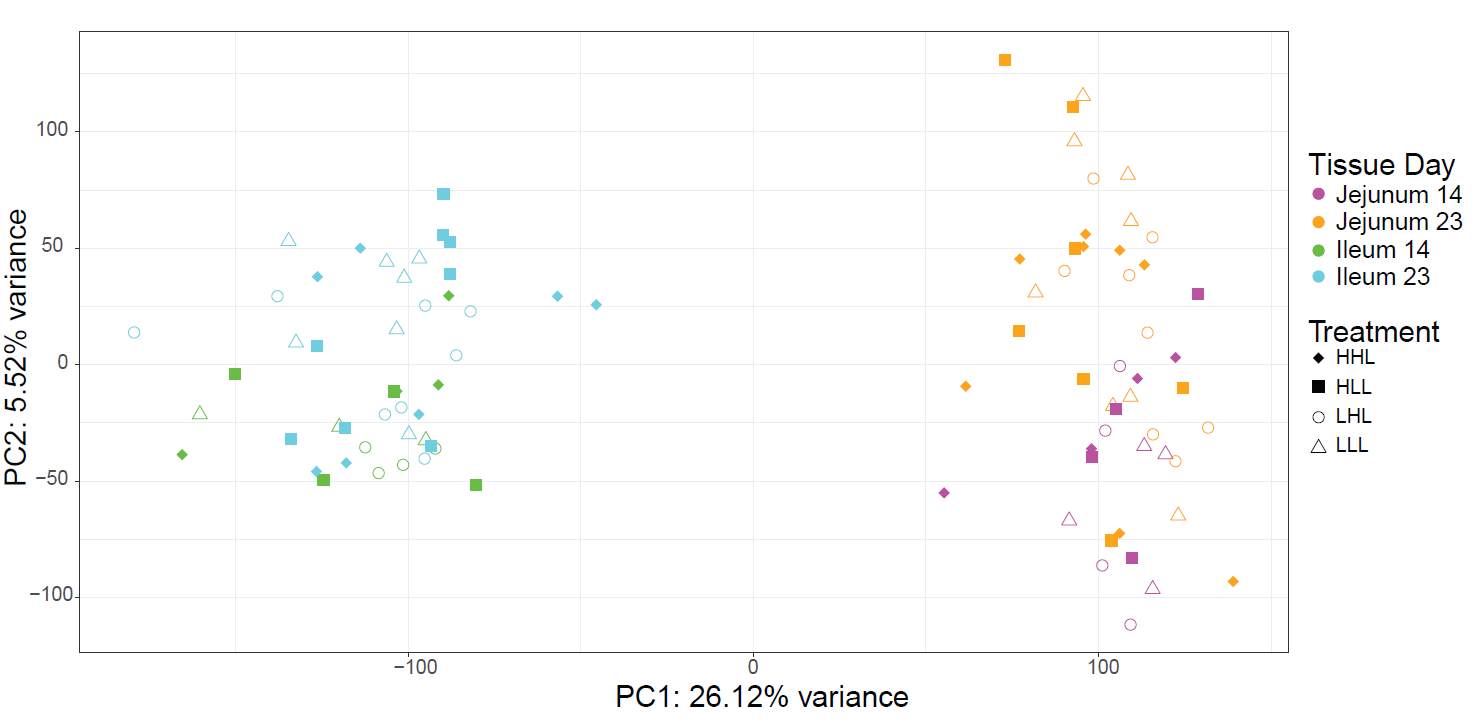


**Fig. S7** Principal Component Analysis of pig gene expression in both jejunum and ileum together at d 14 and 23 per treatment group (2_LLL, 2_LHL, 2_HLL, and 2_HHL) of study 2. Each symbol represents a sample, where the shape represents the treatment, i.e. diamonds represent 2_HHL, squares represent 2_HLL, open circles represent 2_LHL, and open triangles represent 2_LHL. The color indicates the day for a specific tissue, i.e. for jejunum purple is d 14 and orange is d 23, whereas for ileum green is d 14 and cyan is d 23. Principal Component (PC)1 explains 26.12% of the variance, whereas PC2 explain 5.52% of the variance

**
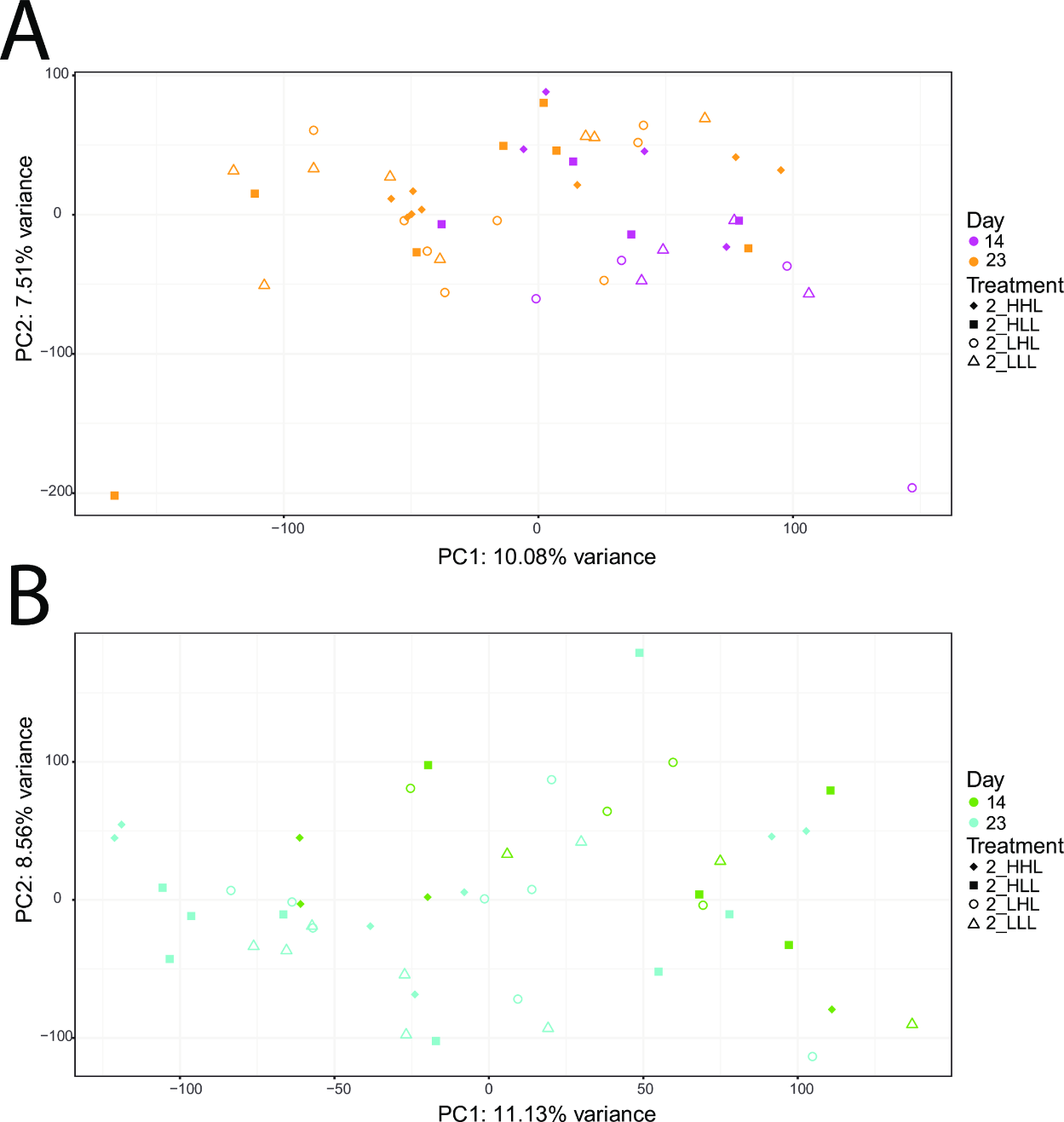
**

**Fig. S8** Principal Component Analysis of gene expression in jejunum and ileum tissue at d 14, and 23 per treatment group (2_LLL, 2_LHL, 2_HLL and 2_HHL)**.** Panel **A** shows the gene expression of jejunum, where each colour represents a day; purple is d 14, orange is d 23, and red is d 35. Symbols depict the treatment, i.e. diamonds represent 2_HHL, squares represent 2_HLL, open circles represent 2_LHL, and open triangles represent 2_LHL. Principal Component (PC)1 explains 10.08% of the variance, whereas PC2 explain 7.51% of the variance. Panel **B** shows the gene expression of ileum, where each colour represents a day; green is d 14, cyan is d 23, and blue is d 35. Symbols depict the treatment, i.e. diamonds represent 2_HHL, squares represent 2_HLL, open circles represent 2_LHL, and open triangles represent 2_LHL. Principal Component (PC)1 explains 11.13% of the variance, whereas PC2 explain 8.56% of the variance

**Table S1** Calculated ingredient and nutrient composition of the weaning and control experimental starter diets (g/kg) of study 1

|  | **Weaning** |  |  | **Starter^1^** |  |
| --- | --- | --- | --- | --- | --- |
| Wheat | 145.7 |  |  | 198.4 |  |
| Barley | 300.0 |  |  | 300.0 |  |
| Maize | 200.0 |  |  | 200.0 |  |
| Whey powder | 75.0 |  |  | - |  |
| Soybean meal | 100.0 |  |  | 200.0 |  |
| Soya concentrate | 30.0 |  |  | - |  |
| Potato protein | 20.0 |  |  | - |  |
| Sunflower meal | 30.0 |  |  | 30.0 |  |
| Linseed | 30.0 |  |  | 10.0 |  |
| Soya oil | 30.0 |  |  | 22.0 |  |
| Premix^2^ | 5.0 |  |  | 5.0 |  |
| Chalk | 6.5 |  |  | 14.0 |  |
| Monocalcium phosphate | 8.0 |  |  | 8.5 |  |
| NaCl | 5.0 |  |  | 5.5 |  |
| Natuphos 5000G | 0.10 |  |  | 0.10 |  |
| Ca-formiate | 7.50 |  |  | - |  |
| *L*-lysine HCl | 4.20 |  |  | 4.00 |  |
| *DL*-methionine | 1.50 |  |  | 1.20 |  |
| *L*-Threonine | 1.10 |  |  | 1.10 |  |
| *L*-Tryptophan | 0.40 |  |  | 0.20 |  |
|  |  |  |  |  |  |
| Dry matter | 889 |  |  | 879 |  |
| Crude protein | 177 |  |  | 180 |  |
| Crude fat (EE) | 59 |  |  | 46 |  |
| Ash | 52 |  |  | 55 |  |
| Crude fibre | 36 |  |  | 38 |  |
| Starch (Ewers) | 375 |  |  | 409 |  |
| Sugars | 80 |  |  | 36 |  |
| NE, MJ/kg | 10.04 |  |  | 9.62 |  |
| Calcium | 7.7 |  |  | 8.0 |  |
| P (total) | 5.6 |  |  | 5.6 |  |
| P (dig.) | 3.7 |  |  | 3.5 |  |
| Sodium | 2.5 |  |  | 2.2 |  |
| Potassium | 8.1 |  |  | 8.0 |  |
| Chloride | 5.8 |  |  | 4.5 |  |
| Base-excess, meq/kg | 152 |  |  | 172 |  |
|  |  |  |  |  |  |
| Amino acids | Total | AID^3^ |  | Total | AID^3^ |
| Lysine | 12.0 | 10.5 |  | 11.5 | 10.0 |
| Methionine | 4.4 | 4.0 |  | 4.0 | 3.5 |
| Cysteine | 3.1 | 2.4 |  | 3.1 | 2.4 |
| Methionine + Cysteine | 7.5 | 6.4 |  | 7.1 | 6.0 |
| Threonine | 7.8 | 6.3 |  | 7.4 | 5.9 |
| Tryptophan | 2.5 | 2.0 |  | 2.3 | 1.9 |
| Isoleucine | 7.3 | 6.1 |  | 7.2 | 5.9 |
| Valine | 8.6 | 6.9 |  | 8.3 | 6.7 |

^1^The high zinc starter diet was formulated by supplementing Zn as ZnO to obtain a diet with 2,690 mg Zn/kg

^2^Both the weaning and the starter diets contained 20 mg/kg supplemented Cu. The vitamin-mineral premix supplied per kg feed: 10,000 IU vitamin A, 2,000 IU vitamin D_3_, 40 mg vit E, 1.5 mg vitamin K, 1.0 mg vitamin B_1_, 4 mg vitamin B_2_, 15 mg d-pantothenic acid, 30 mg niacine, 50 µg biotine, 20 µg vitamin B_12_, 0.4 folium acid, 1.5 mg vitamin B_6_, 150 mg choline chloride, 100 mg Fe, 20 mg Cu, 70 mg Zn, 30 mg Mn, 0.7 mg I, 0.25 mg Se, 125 mg Oxytrap PXN (antioxidant)

^3^AID: apparent ileal digestible

**Table S2** Analysed nutrient composition (g/kg dry matter) of the experimental diets provided during d14-35 of study 1

|  | **Low Zinc** | **High Zinc** |
| --- | --- | --- |
| Zinc, mg/kg | 100 | 2,690 |
| Dry matter | 888 | 888 |
| Ash | 55 | 54 |
| Crude protein (N × 6.25) | 181 | 182 |
| Crude fibre | 37 | 36 |
| Sugars | 40 | 39 |
| Crude fat (EE) | 49 | 48 |
| Starch (Ewers) | 419 | 417 |

**Table S3** Calculated ingredient and nutrient composition of the low zinc weaner diet and the low zinc starter diet (g/kg) of study 2

|  | **Weaning** |  |  | **Starter^1^** |  |
| --- | --- | --- | --- | --- | --- |
| Wheat | 150.2 |  |  | 203.5 |  |
| Barley | 300.0 |  |  | 300.0 |  |
| Maize | 200.0 |  |  | 200.0 |  |
| Whey powder | 75.0 |  |  |  |  |
| Soybean meal | 95.0 |  |  | 195.0 |  |
| Soya concentrate | 30.0 |  |  |  |  |
| Potato protein | 20.0 |  |  |  |  |
| Sunflower meal | 30.0 |  |  | 30.0 |  |
| Linseed | 30.0 |  |  | 10.0 |  |
| Soya oil | 30.0 |  |  | 21.5 |  |
| Premix^2^ | 5.0 |  |  | 5.0 |  |
| Chalk | 6.5 |  |  | 14.2 |  |
| Monocalcium phosphate | 8.0 |  |  | 8.5 |  |
| NaCl | 4.0 |  |  | 5.5 |  |
| Sodium bicarbonate | 1.3 |  |  |  |  |
| Natuphos 5000G | 0.1 |  |  | 0.1 |  |
| Ca-formiate | 7.5 |  |  |  |  |
| *L*-Lysine HCl | 4.2 |  |  | 4.0 |  |
| *DL*-Methionine | 1.5 |  |  | 1.2 |  |
| *L*-Threonine | 1.1 |  |  | 1.1 |  |
| *L*-Tryptophan | 0.6 |  |  | 0.4 |  |
|  |  |  |  |  |  |
| Dry matter | 890 |  |  | 879 |  |
| Crude protein | 177 |  |  | 181 |  |
| Crude fat (EE) | 59 |  |  | 45 |  |
| Ash | 52 |  |  | 55 |  |
| Crude fibre | 35 |  |  | 37 |  |
| Starch (Ewers) | 377 |  |  | 412 |  |
| Sugars | 79 |  |  | 35 |  |
| NE, MJ/kg | 10.06 |  |  | 9.63 |  |
| Calcium | 7.7 |  |  | 8.0 |  |
| P, total | 5.7 |  |  | 5.7 |  |
| P dig. | 3.7 |  |  | 3.5 |  |
| Sodium | 2.5 |  |  | 2.2 |  |
| Potassium | 7.9 |  |  | 7.9 |  |
| Chloride | 5.2 |  |  | 4.5 |  |
| Zn^4^ | 27 |  |  | 30 |  |
| Base-excess, meq/kg | 164 |  |  | 168 |  |
|  |  |  |  |  |  |
| Amino acids | Total | AID^3^ |  | Total | AID^3^ |
| Lysine | 11.9 | 10.5 |  | 11.5 | 10.0 |
| Methionine | 4.4 | 4.0 |  | 4.0 | 3.6 |
| Cysteine | 3.1 | 2.4 |  | 3.2 | 2.4 |
| Methionine + Cysteine | 7.5 | 6.4 |  | 7.1 | 6.0 |
| Threonine | 7.8 | 6.3 |  | 7.5 | 5.9 |
| Tryptophan | 2.7 | 2.2 |  | 2.5 | 2.1 |
| Isoleucine | 7.3 | 6.1 |  | 7.2 | 5.9 |
| Valine | 8.5 | 6.9 |  | 8.3 | 6.7 |

^1^The high zinc weaner and starter diets were formulated by supplementing Zn as ZnO to obtain diets with 2730 and 2850 mg Zn per kg, respectively
^2^Both the weaning and the starter diets contained 20 mg/kg supplemented Cu. The vitamin-mineral premix supplied per kg feed: 10,000 IU vitamin A, 2,000 IU vitamin D_3_, 40 mg vit E, 1.5 mg vitamin K, 1.0 mg vitamin B_1_, 4 mg vitamin B_2_, 15 mg d-pantothenic acid, 30 mg niacine, 50 µg biotine, 20 µg vitamin B_12_, 0.4 folium acid, 1.5 mg vitamin B_6_, 150 mg choline chloride, 100 mg Fe, 20 mg Cu, 70 mg Zn, 30 mg Mn, 0.7 mg I, 0.25 mg Se, 125 mg Oxytrap PXN (antioxidant)

^3^AID: apparent ileal digestible

^4^Zn contribution from the feed ingredients, excluding the contribution of Zn in the premix (70 mg/kg)

**Table S4** Analysed nutrient composition (g/kg) of the experimental weaning (d 0–14) and starter diets (d 14–35) of study 2

|  | **Weaning** |  | **Starter** |  |
| --- | --- | --- | --- | --- |
|  | **Low Zinc** | **High Zn** | **Low Zinc** | **High Zn** |
| Zinc, mg/kg | 121 | 2730 | 142 | 2850 |
| Dry matter | 892 | 894 | 885 | 885 |
| Ash | 59 | 62 | 64 | 68 |
| Crude protein | 173 | 174 | 183 | 184 |
| Crude fibre | 35 | 35 | 36 | 39 |
| Total sugars (gluc. equiv.) | 64 | 65 | 40 | 39 |
| Crude fat (EE) | 64 | 63 | 51 | 49 |
| Starch (Ewers) | 374 | 372 | 408 | 402 |
| Sodium | 3.2 | 3.2 | 3.2 | 3.3 |
| Phosphorus | 6.0 | 5.9 | 6.0 | 5.9 |
| Calcium | 10.6 | 10.4 | 11.4 | 11.3 |

**Table S5** Comparison per treatment of body weight (BW) on d −1, 14, 23 and 35, body weight gain (BWG), feed intake and feed conversion ratio (FCR) of the pigs over the periods d 0–14, 14–23, 23–35 and d 0–35 in study 1

|  |  | **1_LLL** | **1_LHL** | ***P*-value** |
| --- | --- | --- | --- | --- |
| BW, kg | d −1 | 7.9 |  |  |
|  | d 14 | 10.9 |  |  |
|  | d 23 | 14.5 | 14.8 | 0.83 |
|  | d 35 | 20.7 | 20.1 | 0.75 |
| BWG, g/d | d 0–14 | 215 |  |  |
|  | d 14–23 | 400 | 440 | **0.05** |
|  | d 23–35 | 524 | 475 | 0.27 |
|  | d 0–35 | 368 | 362 | 0.83 |
| Feed intake, g/d | d 0–14 | 264 |  |  |
|  | d 14–23 | 575 | 609 | 0.38 |
|  | d 23–35 | 771 | 709 | 0.35 |
|  | d 0–35 | 517 | 506 | 0.79 |
| FCR | d 0–14 | 1.23 |  |  |
|  | d 14–23 | 1.44 | 1.39 | 0.52 |
|  | d 23–35 | 1.47 | 1.49 | 0.60 |
|  | d 0–35 | 1.40 | 1.40 | 0.77 |

**Table S6** Zinc concentrations in blood plasma on d 14, 23 and 35 of animals in study 1, μmol/mL

|  | **d 14^*^** | **d 23** | **d 35** |
| --- | --- | --- | --- |
| **1_LLL** | 17.1 (± 3.4) | 17.3 (± 2.3) | 16.7 (± 1.9) |
| **1_LHL** | 17.7 (± 0.5) | 32.5 (± 14.1) | 19.0 (± 1.4) |
| ***P*^2^** | 0.76 | 0.03 | 0.04 |

^1^Student’s *t*-test (2-tailed, homoscedastic)

^*^Technically these are the same treatment group, but are already assigned to a (future) treatment group

**Table S7** Body weight on d 0, 14, 23 and 35, body weight gain (BWG), feed intake and feed conversion ratio (FCR) over the periods d 0–14, 14–23, and 23–35 in study 2

|  | **Day** | **2_LLL + 2_LHL*** | | **2_HLL + 2_HHL^*^** | | | | ***P*-value** | | | **LSD** | |  |  |
| --- | --- | --- | --- | --- | --- | --- | --- | --- | --- | --- | --- | --- | --- | --- |
| BW, kg | 0 | 7.55 | | 7.55 | | | | 1 | | | 0.89 | |  |  |
| BW, kg | 14 | 10.6 | | 11.2 | | | | 0.33 | | | 1.2 | |  |  |
| BWG, g/d | 0–14 | 221 | | 262 | | | | **0.01** | | | 31 | |  |  |
| Feed intake, g/d | 0–14 | 297 | | 344 | | | | **0.04** | | | 45 | |  |  |
| FCR | 0–14 | 1.34 | | 1.31 | | | | 0.44 | | | 0.09 | |  |  |
|  | | | | | | | | |  | |  |  |  |  |
|  | **Day** | **2_LLL** | **2_LHL** | | **2_HLL** | **2_HHL** | ***P*-value** | | | **LSD** | |  |  |  |
| BW, kg | 23 | 14.3 | 14.4 | | 14.4 | 15.2 | 0.11 | | | 0.87 | |  |  |  |
| BWG, g/d | 14–23 | 429 | 417 | | 383 | 444 | 0.38 | | | 76 | |  |  |  |
| Feed intake, g/d | 14–23 | 532 | 553 | | 512 | 580 | 0.33 | | | 80 | |  |  |  |
| FCR | 14–23 | 1.25 | 1.33 | | 1.34 | 1.31 | 0.27 | | | 0.11 | |  |  |  |
| BW, kg | 35 | 20.1 | 20.5 | | 20.6 | 21.0 | 0.82 | | | 2.2 | |  |  |  |
| BWG, g/d | 23–35 | 495 | 523 | | 515 | 490 | 0.88 | | | 108 | |  |  |  |
| Feed intake, g/d | 23–35 | 806 | 793 | | 803 | 772 | 0.95 | | | 142 | |  |  |  |
| FCR | 23–35 | 1.63 | 1.52 | | 1.59 | 1.57 | 0.45 | | | 0.15 | |  |  |  |
| BWG, g/d | 0–35 | 364 | 380 | | 377 | 391 | 0.78 | | | 58 | |  |  |  |
| Feed intake, g/d | 0–35 | 532 | 533 | | 544 | 552 | 0.93 | | | 79 | |  |  |  |
| FCR | 0–35 | 1.46 | 1.40 | | 1.45 | 1.41 | 0.14 | | | 0.06 | |  |  |  |

^*^Here we combine the groups because at d 14 only 2 biological groups exist, i.e., Low and High ZnO

**Table S8** Zinc concentrations (μmol/mL) in blood plasma on d 14, 23 and 35 in study 2

| **Treatment** | **2_LLL + 2_LHL** |  | **2_HLL + 2_HHL** |  | **LSD** | ***P*** |
| --- | --- | --- | --- | --- | --- | --- |
| 14 | 10.3^a^ |  | 22.8^b^ |  | 2.8 | < 0.001 |
|  | | | | | | |
| **Treatment** | **2_LLL** | **2_LHL** | **2_HLL** | **2_HHL** | **LSD** | ***P*** |
| 14 | 10.9^a^ | 9.8^a^ | 22.2^b^ | 23.5^b^ | 4.1 | 0.001 |
| 23 | 16.9^a^ | 23.1^b^ | 17.6^a^ | 27.2^c^ | 6.0 | 0.006 |
| 35 | 15.3 | 16.0 | 15.9 | 18.3 | 2.9 | 0.190 |
| ^a–c^Values with a different superscript differ at *P* < 0.05 | | | | |  |  |
